# Controlling the White‐Light Generation of [(RSn)_4_E_6_]: Effects of Substituent and Chalcogenide Variation

**DOI:** 10.1002/anie.201909981

**Published:** 2019-10-15

**Authors:** Eike Dornsiepen, Florian Dobener, Sangam Chatterjee, Stefanie Dehnen

**Affiliations:** ^1^ FB Chemie and Wissenschaftliches Zentrum für Materialwissenschaften (WZMW) Philipps-Universität Marburg Hans-Meerwein-Straße 4 35043 Marburg Germany; ^2^ Institute of Experimental Physics I Justus Liebig University Gießen and Center for Materials Research (ZfM) Heinrich-Buff-Ring 16 35392 Gießen Germany

**Keywords:** main-group clusters, quantum chemical calculations, second-harmonic generation, substituent effects, white-light generation

## Abstract

Adamantane‐type organotin chalcogenide clusters of the general composition [(RT)_4_S_6_] (R=aromatic substituent, T=Si, Ge, Sn) have extreme non‐linear optical properties that lead to highly directional white‐light generation (WLG) upon irradiation with an IR laser diode. However, the mechanism is not yet understood. Now, a series of compounds [(RSn)_4_E_6_] (R=phenyl, cyclopentadienyl, cyclohexyl, benzyl, CH_2_CH_2_(C_6_H_4_)CO_2_Et; E=S, Se), were prepared, characterized, and investigated for their nonlinear optical properties. With the exception of crystalline [(BnSn)_4_S_6_], all these compounds exhibit WLG with similar emission spectra; slight blue‐shifts are observed by introduction of cyclopentadienyl substituents, while the introduction of Se in the inorganic core can provoke a red‐shift. These investigations disprove the initial assumption of an aromatic substituent being a necessary precondition; the precondition seems to be the presence of (cyclic) substituents providing enough electron density.

## Graphical abstract

**In search of the preconditions** for the generation of highly directed white light by means of amorphous molecular materials of the general formula [(RT)_4_E_6_] (R=organic group, T/E=Group 14/16 elements), nine new compounds were studied. Surprisingly, R does not need to be an aromatic ligand, in striking contrast to previous assumptions.

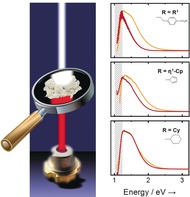

## Introduction

Organo Group 14 chalcogenide clusters are organic/inorganic hybrid compounds consisting of an inorganic cage, which are derived from chalcogenido Group 14 anions^1^, ^2^ that feature an organic substituent shell attached to the core by covalent Group 14 element–carbon bonds. Chalcogenido Group 14 anion salts have been actively investigated owing to their tunable optoelectronic and semiconducting properties, resulting in diverse applications.^3^, ^4^, ^5^, ^6^ The decoration of such cluster anions with organic substituents not only allows the isolation of compounds with discrete, neutral cluster molecules, it also enables further tailoring of material properties such as solubility in organic solvents or reactivity towards organic molecules, metal salts, or surfaces.^7^

In the context of our investigation of functionalized organo Group 14 chalcogenide clusters,^8^, ^9^, ^10^, ^11^, ^12^ we have recently found an unprecedented, extreme non‐linear optical behavior of the styryl‐decorated cluster [(StySn)_4_S_6_] (Sty=4‐vinylphenyl),^13^ which belongs to the family of organo Group 14 sesquichalcogenide clusters of the general formula [(RT)_4_E_6_] (R=organic substituent; T=Si, Ge, Sn; E=O, S, Se, Te).^14^, ^15^, ^16^, ^17^, ^18^ [(StySn)_4_S_6_] has been obtained as an amorphous powder, but DFT calculations reveal that the adamantane‐type structure are favored over the double‐decker‐type isomer by ca. 30 kJ mol^−1^. While compounds that lack inversion symmetry are widely known for second‐harmonic generation (SHG), we observed white‐light generation (WLG) upon irradiation with a commercially available continuous wave (CW) near‐infrared laser diode.^13^ This unexpected phenomenon was intuitively attributed to the amorphous nature of the compound. In the respective powders, SHG is clearly prohibited by the random orientation of the molecules, which, at the same time, leads to an extreme broadening of the SHG emission. Yet, the exact mechanism of this non‐linear response is still unknown. Therefore, we are currently about to expand the library of related compounds for getting more insight in the new WLG behavior.

We have furthermore prepared a range of adamantane‐type clusters with different organic substituents and Group 14 elements: [(PhSn)_4_S_6_] and [(PhGe)_4_S_6_] exhibit WLG, while [(PhSi)_4_S_6_], [(MeSn)_4_S_6_] and [(NpSn)_4_S_6_] do not emit white light; yet, these compounds still show non‐linear optical properties by strong SHG instead.^19^ For [(PhSi)_4_S_6_], this is easily attributed to the crystallinity of the compound, which suppresses WLG. In the case of [(MeSn)_4_S_6_], the organic substituent shell does not contain an aromatic π‐electron system; as [(MeSn)_4_S_6_] lacks any evidence for long‐range order but shows SHG, the presence of π‐aromatic substituent molecules was intuitively assumed to be a necessary condition for WLG. The lack of WLG observed for the naphthyl‐substituted [(NpSn)_4_S_6_] was attributed to beginning long‐range order in the amorphous powder due to π‐stacking.^19^

Although the assumption of an aromatic π‐electron system as a critical parameter seemed plausible, its definite role has remained unclear so far. So, we intend to further narrow down the chemical and electronic pre‐conditions for WLG and have designed a new series of compounds that have cyclic substituents R, hence including non‐aromatic ones. Herein, we report on their synthesis and characterization as well as on the non‐linear optical response that was shown this way to be feasible with cyclic substituents lacking aromaticity, as well.

Clusters of the type [(RSn)_4_E_6_] (R=Ph, Bn, CH_2_CH_2_(C_6_H_4_)CO_2_Et, η^1^‐Cp, Cy; E=S, Se) have been prepared according to Equation (1) and fully characterized.

(1) $$\mathrm{RSnCl}{}_{3}\overset{\left(\mathrm{Me}{}_{3}\mathrm{Si}\right){}_{2}E}{\underset{\mathrm{toluene}}{\to }}\left[\left(\mathrm{RSn}\right){}_{4}E{}_{6}\right]$$

**Scheme 1:**
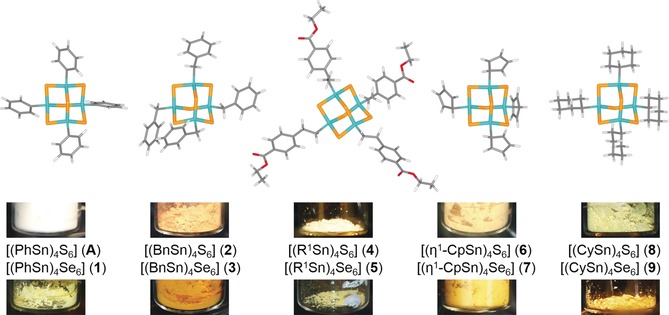
Synthesis of compounds **1**–**9** (Ph=phenyl, Bn=benzyl, R^1^=CH_2_CH_2_(C_6_H_4_)CO_2_Et, η^1^‐Cp=η^1^‐cyclopentadienyl, Cy=cyclohexyl), along with DFT‐optimized molecular structures, shown for the selenide clusters as examples, and photographs of samples of the isolated compounds.

The subtle influence of the substituent was probed by introducing either non‐aromatic cyclic substituents or an aliphatic linker to further separate the aromatic ring from the cluster core, or finally additional functionalization of the aromatic substituent. To gain further insight into the role of the cluster core's composition, the series of compounds is realized for both E=sulfur and selenium, with the latter being the first selenide clusters of this kind to be examined for non‐linear optical properties. Scheme 1 summarizes all compounds that were synthesized and studied herein.

## Results and Discussion

With the chosen series of compounds, we intend to gain more insight into the prerequisites for WLG^20^ by answering the following questions:

1) Is the emission spectrum affected by replacement of sulfur with selenium; 2) does the aromatic substituent need to be bonded directly to the inorganic core, or is WLG also observed if an aliphatic spacer is inserted between the cluster core and the π‐system; 3) is a π‐aromatic substituent necessary at all, or do π‐electrons located in isolated double‐bonds also lead to the observation of WLG; and 4) are substituents with any kind of π‐electrons generally needed for WLG, or can molecules that bear fully saturated organic substituents also exhibit WLG.

**Figure 1:**
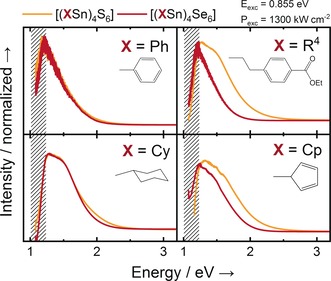
WLG emission spectra of organotin sulfide and selenide clusters (from top left to bottom right): **A** and **1** (R=phenyl), **4** and **5** (R=R^1^=CH_2_CH_2_(C_6_H_4_)CO_2_Et), **6** and **7** (R=η^1^‐cyclopentadienyl), **8** and **9** (R=cyclohexyl). Each spectrum is response‐corrected with a calibrated tungsten halogen lamp. In the cross‐hatched area (>1000 nm) the instrument response of the used silicon charge‐coupled device array detector (cooled down to −60 °C) is very weak, hence inhibiting a reliable spectral intensity correction in this range.

Upon reaction of the corresponding organotin trichlorides and (Me_3_Si)_2_E in toluene, the compounds precipitate from solution and are isolated by filtration. Amorphousness versus crystallinity is then probed by powder X‐ray diffraction (Supporting Information, Figures S42–S50), and emission spectra of all new compounds are collected. The emission spectra of the amorphous compounds **1** and **4**–**9** are shown along with the spectrum of known compound [(PhSn)_4_S_6_] (**A**) in Figure 1.

To address question 1, we compare the emission spectra of the sulfide clusters with those of the selenide analogues. The observations will be discussed at each of the homologous pairs along this report and summarized at the end. In case of compounds [(PhSn)_4_S_6_] (**A**) and [(PhSn)_4_Se_6_] (**1**), we do not see a significant change in the shape of the emission, thus concluding that the WLG is largely independent from the composition of the inorganic core here. This is similar to what has been reported for the variation of the Group 14 element before.^19^

Regarding question 2, we investigate the benzyl‐substituted clusters **2** and **3**, and also explored compounds **4** and **5**. The latter feature a CH_2_CH_2_(C_6_H_4_)CO_2_Et substituent in which the phenyl moiety is separated from the tin atom by an CH_2_ unit. Compounds **2** and **3** show a striking difference from the previously prepared clusters. While all other compounds are obtained as amorphous powders, **2** and **3** are isolated in crystalline form suitable for single‐crystal structural analysis.

Apparently, the methylene groups in the benzyl substituents give the molecules the necessary flexibility to orient in a way that allows for an efficient packing in a crystal lattice. Hence, although enhanced flexibility of substituents often disturbs long‐range order, and also leads to the remarkably amorphous powders of most of the clusters discussed herein, the benzyl‐substituents seem to lead to an opposite effect for a gain of lattice energy or alternate second‐order interaction energy. So far, we have identified three possible ways to do so in this class of compounds: 1) beginning long‐range order if the aromatic system is large enough for efficient π‐stacking (as found for naphthyl substituents in [(NpSn)_4_S_6_]),^19^ 2) a relatively small size of the cluster core relative to (small) aromatic substituents, which in the sum allows for a sufficiently close approach of the clusters for efficient π‐stacking (as found for [(PhSi)_4_S_6_]),^19^ 3) setting apart a (small) aromatic group from the cluster core by introducing an organic spacer group (as found for **2** and **3**). Although we did not expect crystalline compounds **2** and **3** to generate white light, we investigate their structures, and also their non‐linear optical response.

**Figure 2:**
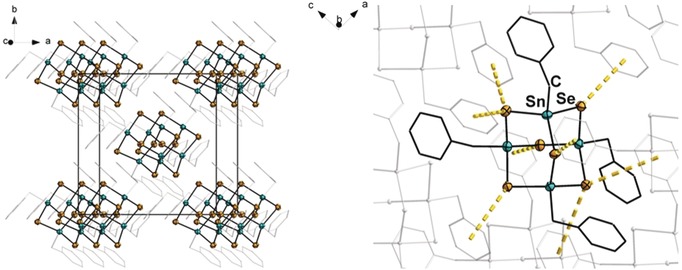
Packing of the cluster molecules in the crystal structure of **3** (left) and illustration of the intermolecular interaction of neighboring clusters (right). Ellipsoids are set at 50 % probability; hydrogen atoms are omitted for clarity. The analogue structure of **2** is given in the Supporting Information, Figure S51.

Compound **2** crystallizes in the tetragonal crystal system in the space group *I* $\bar{4}$ with two formula units per unit cell. The Sn−S bonds show little variation, ranging from 2.3948(18) to 2.4086(19) Å, which is in the usual range for organotinsulfides.^9^ The same holds for the bond angles within the inorganic core (103.60(11)–115.71 (6)°), which are all in close proximity to an ideal tetrahedral angle. The selenide cluster **3** is isostructural to its lighter homologue. The Sn−Se bond lengths are 2.5227(9)–2.5292(10) Å long, thus longer as in **2** and in the expected range for organotinselenides.^21^ The variation in bond angles is slightly greater than in **2**, ranging from 100.82(5) to 117.13(3)°. For both of the compounds, the packing diagrams indicate the absence of π‐stacking in spite of the presence of terminal phenyl rings (see the Supporting Information, Figure S33 for **2**, and Figure 2 for **3**). Instead, the benzyl substituents are well separated from each other. However, the packing of the molecules allows for another type of secondary interactions, which are found between the chalcogenide atoms in the cluster core and the phenyl rings of the neighboring molecules (shortest distance 3.5221(79) Å for **2** and 3.5303(81) Å for **3**).

**Figure 3:**
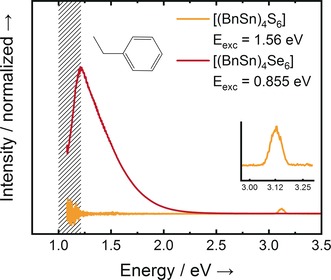
Emission spectra of compounds **2** (no WLG) and **3** (WLG). Two different excitation schemes were employed: a pulsed Ti:sapphire laser excitation and a 1450 nm continuous wave (CW) laser diode. As the field strength of the CW laser diode is not sufficient to generate detectable second harmonic emission from compound **2**, we use the 100 fs pulses from the Ti:sapphire laser to generate a detectable signal. To keep the data comparable to the measurements in Figure 1, we decided to show the signal excited by the CW laser diode of compound **3**. The excitation at 800 nm shows a very similar signal owing to the excitation wavelength‐independent emission process (see also the Experimental Section in the Supporting Information).

The emission spectra of **2** and **3** are shown in Figure 3. While the sulfide cluster **2** shows SHG, as expected for a crystalline compound, the selenide compound **3** unexpectedly featured WLG. Yet, as WLG in the crystalline state is very unlikely to happen for the reasons given above, we also inspect the thermal behavior of both compounds. While **2** starts decomposing in the temperature range of 160–180 °C without melting, **3** already melts at 141 °C without decomposition. So, their different optical behavior might be put down to compound **3** melting under irradiation with the result that the order vanishes, which allows for WLG. To our knowledge, this observation would be the first experimental evidence of WLG to occur in liquids/melts under CW laser irradiation. This is similar to WLG upon in situ amorphization in the solid state, which was observed for a series of unsymmetrically substituted clusters recently.^22^

To allow for the formation of amorphous solids with this kind of substituents, we explore compounds with a larger spacer length, namely compounds **4** and **5** comprising an ethylene group between the cluster core and the phenyl group (see Scheme 1, bottom). We hoped that the even enhanced flexibility of the substituents would inhibit crystallization this time, which indeed was the case. The emission spectra of these compounds are shown in the top right part of Figure 1. Both compounds exhibit WLG. The emission spectrum is not modified significantly in comparison to that of compounds **A** and **1** upon inclusion of a longer spacer. As for the quoted compounds, the emission sets in at energies slightly above those of the exciting laser, and drops off at the energy where SHG would be expected. However, by replacement of sulfur with selenium, we induce a red‐shift of the emission spectrum that was not observed for the phenyl‐substituted clusters, which we cannot explain to date. Yet, to summarize our result regarding question 2, the final answer to question 2 is that in the case of amorphous solids, the aromatic substituents may be separated from the inorganic cluster core. Having learned that aliphatic spacers do not inhibit WLG in principle, we investigate if an aromatic π electron system is at all a necessary prerequisite for WLG. For this purpose, we prepare compounds with localized π‐electrons in a non‐aromatic system, namely the η^1^‐cyclopentadienyl‐substituted clusters **6** and **7**. As shown in Figure 1 (bottom right), these compounds also show WLG, thus rendering the essential need for aromatic substituents obsolete. However, in both cases, we observed a steeper drop‐off at the high‐energy edge of the spectrum, resulting in a larger blue portion of the emitted light. As for the other homologous pairs discussed above, a red‐shift is observed upon inclusion of selenium into the inorganic core of **7** as compared to the spectrum measured for **6**.

The last logical step in this study is to completely dispense with π‐electrons in the substituents. As we knew from our previous studies that simple aliphatic substituents like methyl or butyl do not lead to WLG, but cause SHG instead, we probe the effect of a saturated, yet cyclic substituent. The substituent of choice here is cyclohexyl for its relationship with the phenyl group regarding the number of C atoms, which was realized in compounds **8** and **9** (Figure 1, bottom left). We observe WLG for both of these compounds, so our conclusion it that the WLG cannot be based on an electronic excitation of π electrons. In this case, we did not observe a red‐shift between the spectrum of the sulfide cluster **8** and that of the selenide cluster **9**.

All these results together clearly show the explanation for the observed phenomenon is to be refined as follows: The WLG process is based upon excitation of electrons near the Fermi level with photon energies below the HOMO–LUMO gap. Hence, from all that we have learned in this work, the crucial requirement with respect to the electronic structure of potential white‐light emitters is a sufficiently large HOMO–LUMO gap. Otherwise, electronic excitations into states with much longer lifetimes gain the upper hand, in which case other relaxation pathways like photoluminescence will be observed. Additionally, the amorphous nature of the compounds is key in deciding whether WLG or SHG is observed, because crystallinity allows for a preference of SHG emission.

**Figure 4:**
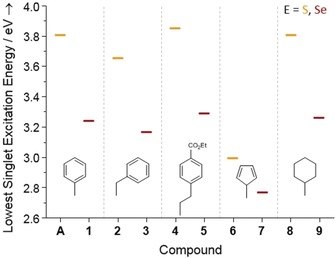
Lowest singlet excitation energies for compounds **A**, and **1**–**9**, as calculated by means of TD‐DFT studies. The nature of the organic substituent is shown; S versus Se ligands are indicated by yellow or red bars, respectively.

To confirm the requirement of sufficiently large HOMO–LUMO gaps, we inspect the electronic absorption properties of the compounds by means of time‐dependent DFT (TD‐DFT) calculations. The lowest singlet excitation energies are given in Figure 4 and the Supporting Information, Table S7. Figures S53–S57 show the corresponding absorption spectra.

In all cases, the HOMO–LUMO gaps are larger than the highest emission energies, in agreement with the statement above. Furthermore, the gaps of selenide clusters are calculated to be smaller than those of the corresponding sulfides. This correlates well with the different colors of the products, which are colorless or yellow in case of the sulfur compounds, and darker (yellow to dark orange, see Scheme 1) in case of the corresponding selenides.

Yet, this does not explain the red‐shift in the emission spectra observed for compounds **5** and **7**, as the emission in both cases is limited at an energy of about 2.0 eV, which is still significantly below the lowest excitation energies. Moreover, to this date, we cannot explain why the substitution of selenium for sulfur induces a red‐shift of the emission in some cases, while in some cases no such effect occurs. This will be subject to ongoing studies within the framework of FOR 2824.

## Conclusion

We report on a study that provides a big step forward in the understanding of this class of white‐light emitters based on adamantane‐type organotin chalcogenide clusters. While the previously stated precondition of the materials to be amorphous was substantiated, our new findings prompted us to re‐phrase some of our former assumptions that were made based on a much smaller cohort of compounds. Clusters of the type [(RSn_4_E_6_)] are capable of white‐light generation (WLG) if they fulfill the following preconditions: The materials need to contain electron‐rich (cyclic) substituents, which may (but do not need to) possess π‐electrons, and which do not need to be directly bonded to the inorganic cluster core, as long the spacer group does not cause crystallization. A point that could not be clarified so far is the impact of a homologous replacement of sulfur atoms with its heavier congeners on the emission properties, while the effect on the absorption properties is clearly a red‐shift. Here, we need to refer to future comprehensive work into this direction.

## Experimental Section

Detailed syntheses, characterization, and details of spectroscopic techniques are to be found in the Supporting Information.

All synthetic steps were carried out under exclusion of oxygen and moisture by use of standard Schlenk techniques. Phenyltin trichloride,^23^ bis(trimethylsilyl) sulfide^24^ and selenide,^25^ sodium cyclopentadienide,^26^ tetracyclohexyltin,^27^ benzyltributyltin,^28^ 4‐vinylethylbenzoate,^29^ and tricyclohexylstannane^30^ were prepared according to previously procedures; SnCl_4_ and AIBN were used as received from abcr.

For the synthesis and characterization of BnSnCl_3_ (**A**), R^1^SnCy_3_ (**B**), R^1^SnCl_3_ (**C**), CySnCl_3_ (**D**), and compounds [(PhSn)_4_Se_6_] (**1**), [(BnSn)_4_S_6_] (**2**), [(BnSn)_4_Se_6_] (**3**), [(R^1^Sn)_4_S_6_] (**4**), [(R^1^Sn)_4_Se_6_] (**5**), [(CpSn)_4_S_6_] (**6**), [(CpSn)_4_Se_6_] (**7**), [(CySn)_4_S_6_] (**8**), and [(CySn)_4_Se_6_] (**9**), see the Supporting Information.

Powder X‐ray diffraction patterns were measured on a StadiMP diffractometer by Stoe equipped with a Mythen 1 K silicon strip detector and a Cu‐Kα (*λ*=1.54056 Å) X‐ray source. Samples were measured in transmission between two layers of Scotch Tape (3M).

Data for the single‐crystal X‐ray diffraction analyses were collected on a STOE STADIVARI four‐circle diffractometer using Cu_Kα_ radiation (*λ*=1.54186 Å) at 100 K. Reflection data were processed with X‐Area 1.77.^31^ Structure solution was performed by direct methods and full‐matrix‐least‐squares refinement against *F* ^*2*^ using SHELXT^32^ and SHELXL‐2014^33^ software. CCDC https://www.ccdc.cam.ac.uk/services/structures?id=doi:10.1002/anie.201909981 (**3**) contain the supplementary crystallographic data for this paper. These data are provided free of charge by http://www.ccdc.cam.ac.uk/.

White‐light emission and SHG were performed using a confocal microscopy spectroscopy setup. For excitation, we use either 100‐fs pulses from a titan‐sapphire laser oscillator operating at a repetition rate of 78 MHz tuned to 1.56 eV for SHG experiments and a multi‐mode continuous‐wave diode laser at a photon energy of 0.855 eV for white‐light emission. A 0.5 NA Schwarzschild objective focusses and collects the light onto and from the sample, which is held in vacuum at room temperature (293 K). A lens focuses the back‐reflected light onto an either an imaging camera or the entrance slit of a quarter meter Czechy–Turner spectrometer equipped with a thermoelectrically cooled silicon deep‐depletion charge‐coupled‐device camera.

The apparently different overall shape of the emission spectra in comparison with those reported in former work^14^, ^20^ are due to a detection‐based change and not due to the different excitation wavelength. In the present work, we use a 1450 nm laser diode to suppress the excitation laser line, which would otherwise appear in the employed silicon‐based detection system. Accordingly, we do not use any filters in front of our detection system and thus, we are able to detect in the full response window of the silicon CCD. In the quoted publications, a 980 nm diode was used, which is filtered from the spectra, yielding the altered shape of the WL emission. However, the actual emission is not affected by the change of excitation wavelength and the different appearance is due to the employed filter set in the detection.

Density functional theory (DFT) calculations were carried out with TURBOMOLE^34^ using def2‐TZVP basis sets^35^ and taking advantage of the multipole‐accelerated resolution‐of the‐identity method.^36^ Structures were optimized with the functional BP86.^37^ Time‐dependent DFT (TD‐DFT) calculations were done employing the B3‐LYP functional.^38^

## Conflict of interest

The authors declare no conflict of interest.

## Supporting information

As a service to our authors and readers, this journal provides supporting information supplied by the authors. Such materials are peer reviewed and may be re‐organized for online delivery, but are not copy‐edited or typeset. Technical support issues arising from supporting information (other than missing files) should be addressed to the authors.

Supplementary
