# Supplementary material for: Controlling the White‐Light Generation of [(RSn)4E6]: Effects of Substituent and Chalcogenide Variation
Source: Angew Chem Int Ed Engl. 2019 Oct 15;58(47):17041–6. doi: 10.1002/anie.201909981 (PMC6899930; doi:10.1002/anie.201909981)
Supplement: Supplementary file 1 — Supplementary [file ANIE-58-17041-s001.pdf]

Angewandte Chemie
Eine Zeitschrift der Gesellschaft Deutscher Chemiker

Supporting Information

Controlling the White-Light Generation of $[(RSn)_4E_6]$: Effects of Substituent and Chalcogenide Variation

*Eike Dornsiepen, Florian Dobener, Sangam Chatterjee, and Stefanie Dehnen**

anie_201909981_sm_miscellaneous_information.pdf

Content

1. Details on Syntheses and Characterization

2. NMR Spectra

3. IR Spectra

4. X-Ray Powder Diffraction

5. Single-crystal X-Ray crystallography of Compound 2

6. Single-crystal X-Ray crystallography of Compound 3

7. Lowest Singlet Excitation Energies

8. UV-Vis Absorption Spectra

1. Details on Syntheses and Characterization

General Analytical Techniques: ^{1}H, ^{13}C, ^{31}P and ^{119}Sn NMR spectroscopy was carried out at 25°C using Bruker DRX 300 MHz and DRX 500 MHz spectrometers. The chemical shifts are given in ppm relative to the residual protons of deuterated solvents for ^{1}H spectra and relative to the solvent signal for ^{13}C spectra. ^{13}C, ^{31}P and ^{119}Sn spectra have been measured with ^{1}H decoupling. Me_4Sn was used as internal standard in ^{119}Sn NMR measurements. HR-ESI mass spectra were acquired with an LTQ-FT Ultra mass spectrometer (Thermo Fischer Scientific). The resolution was set to 100.000. Yields are based on the amount of Sn and refer to the amount of product isolated as single crystals.

Synthesis of $BnSnCl_3$ (A)

$BnSnCl_3$ was prepared by a modified literature procedure.[28] Benzyltributyltin (10.3 mL, 30.0 mmol) was cooled to –30°C. After addition of $SnCl_4$ (3.50 mL, 30.0 mmol), the reaction mixture was warmed to room temperature, dissolved in acetonitrile (25 mL) and stirred for 30 min. *n*-Pentane (20 mL) was injected into the solution, rapidly stirred for 3 min and then removed via PTFE cannula. This extraction was repeated ten times to remove Bu_3SnCl. The solvent was removed from the acetonitrile phase at reduced pressure, the remaining solid washed with *n*-pentane (20 mL) and dried *in vacuo*. **A** (6.59 g, 20.8 mmol, 69%) was obtained as a colorless solid. All analytical data are in agreement with reported data.[28]

Synthesis of R^1SnCy_3 (B)

4-Vinylethylbenzoate (600 mg, 3.38 mmol), tricyclohexylstannane (1.00 g, 2.70 mmol) and AIBN (10 mg, 0.06 mmol) were combined in a Schlenk tube und stirred at a temperature of 110°C for sixteen hours with addition of more AIBN (10 mg, 0.06 mmol) after eight hours. The residue was purified by column chromatography (SiO_2, petrol ether/ethyl acetate 9:1). **B** (909 mg, 1.66 mmol, 62%) was obtained as colorless oil.

Synthesis of R^1SnCl_3 (C)

B (909 mg, 1.66 mmol) was dissolved in *n*-pentane (24 mL). $SnCl_4$ (0.20 mL, 1.66 mmol) was slowly added and the reaction mixture stirred at room temperature for three hours. Acetonitrile (16 mL) was added and the stirring continued for 18 hours. The pentane layer was removed via PTFE cannula and the acetonitrile phase extracted three times with *n*-pentane (5 mL each). After solvent removal from the acetonitrile phase, **C** (490 mg, 1.22 mmol, 73%) was obtained as colorless solid.

^{1}H-NMR ($CDCl_3$, 300 MHz)**:** δ = 1.40 (t, $^{3}J_{HH}$ = 7.1 Hz, 3H, $-CH_3$), 2.62 (t, $^{3}J_{HH}$ = 7.8 Hz, 2H, $-SnCH_2-$), 3.26 (t, $^{3}J_{HH}$ = 7.8 Hz, 2H, $-CH_2-$) 4.38 (q, $^{1}J_{HH}$ = 7.1 Hz, 2H, $-OCH_2$), 7.33 (d, $^{3}J_{HH}$ = 8.3 Hz, 2H, -CH*arom*), 8.04 (d, $^{3}J_{HH}$ = 8.3 Hz, 2H, -CH*arom*) ppm. **^{13}C-NMR** (CDCl3, 75 MHz)**:** δ = 14.0, 30.3, 32.8, 60.9, 127.8, 129.6, 130.3, 144.5, 165.9 ppm. **^{119}Sn-NMR** ($CDCl_3$, 187 MHz)**:** δ = 2 ppm.

Synthesis of [$(PhSn)_4Se_6$] (1)

$(Me_3Si)_2Se$ (1.20 mL, 4.79 mmol) was added at room temperature to a solution of $PhSnCl_3$ (0.50 mL, 3.04 mmol) in toluene (20 mL). After a few seconds, a light yellow precipitate formed that was filtered off after 5 minutes. Washing with toluene (5 mL) and drying *in vacuo* afforded **1** (915 mg, 0.73 mmol, 96%) as a light yellow powder.

^{1}H-NMR (CD_2Cl_2, 300 MHz)**:** δ = 7.53 (m, 12H, H_{ortho} + H_{para}), 7.69 (m, 8H, H_{meta}) ppm. **^{13}C-NMR** (CD_2Cl_2, 75 MHz)**:** δ = 129.5 (C_{arom}), 129.9 (C_{arom}), 131.7 (C_{arom}), 134.3 (C_{arom}) ppm. **^{119}Sn-NMR** (CD_2Cl_2, 112 MHz)**:** δ = –100 ($^{1}J_{SnC}$ = 130Hz, $^{1}J_{SnSe}$ = 683 Hz) ppm. **IR:** $\tilde{\nu}$ = 1573 (w), 1473 (w), 1439 (m), 1327 (w), 1296 (w), 1258 (w), 1887 (w), 1156 (w), 1064 (m), 1016 (w), 996 (m), 963 (w), 905 (w), 842 (w), 721 (s), 688 (s), 613 (w), 463 (w), 437 (s) cm^{-1}. **Elemental Analysis:** found (calcd.): C 23.41 (22.93), H 1.65 % (1.60 %).

Synthesis of [$(BnSn)_4S_6$] (2)

A (1.00 g, 3.16 mmol) was dissolved in toluene (20 mL), $(Me_3Si)_2S$ (1.00 mL, 4.76 mmol) added and the solution stirred at room temperature. After 20 minutes, a colorless precipitate was formed that slowly turned yellow. After 35 minutes, the precipitate was filtered off. Leaving the filtrate standing for one day afforded **2** (280 mg, 0.27 mmol, 34%) as colorless crystals.

^{1}H-NMR (CD_2Cl_2, 300 MHz)**:** δ = 3.13 (s, 8H, $-CH_2-$), 7.14 (m, 12H, H_{ortho} + H_{para}), 7.28 (m, 8H, H_{meta}) ppm. **^{13}C-NMR** (CD_2Cl_2, 75 MHz)**:** δ = 36.9 ($-CH_2-$), 126.8 (C_{arom}), 128.9 (C_{arom}), 129.5 (C_{arom}) ppm. The quaternary carbon atom could not be detected. **^{119}Sn-NMR** (CD_2Cl_2, 112 MHz)**:** δ = 121 ($^{1}J_{SnC}$ = 110 Hz) ppm. **IR:** $\tilde{\nu}$ = 445 (s), 550 (m), 570 (m), 619 (w), 697 (s), 728 (s), 761 (s), 800 (m), 844 (w), 858 (m), 904 (m), 1028 (m), 1049 (m), 1109 (m), 1154 (w), 1179 (w), 1206 (m), 1259 (m), 1319 (w), 1334 (w), 1401 (m), 1451 (m), 1488 (m), 1509 (w), 1578 (w), 1595 (m) cm^{-1}. **Elemental Analysis:** found (calcd.): C 32.42 (32.60), H 2.79 (2.74), S 17.60 % (18.64 %).

Synthesis of [$(BnSn)_4Se_6$] (3)

A (1.00 g, 3.16 mmol) was dissolved in toluene (20 mL), $(Me_3Si)_2Se$ (1.20 mL, 4.79 mmol) added and the solution stirred at room temperature. The solution immediately changed its color from colorless via yellow and orange to dark red, formation of a dark red precipitate occurred after 2-3 min. Filtration after 5 min afforded a clear red solution from which **3** (340 mg, 0.26 mmol, 33%) crystallized as orange blocks after one day.

^{1}H-NMR (CD_2Cl_2, 300 MHz)**:** δ = 3.27 (s, 8H, -CH_2-), 7.16 (m, 12H, H_{ortho} + H_{para}), 7.27 (m, 8H, H_{meta}) ppm. ^{13}C and ^{119}Sn NMR spectra could not be obtained due to the compound's low solubility. . **IR:** $\tilde{\nu}$ = 444 (s), 464 (w), 507 (w), 526 (w), 549 (m), 570 (m), 619 (w), 699 (s), 729 (s), 760 (s), 800 (m), 822 (w), 844 (m), 858 (m), 905 (m), 967 (w), 1029 (m), 1047 (m), 1109 (m), 1154 (w), 1205 (m), 1258 (m), 1319 (w), 1334 (w), 1400 (m), 1451 (m), 1488 (m), 1509 (w), 1588 (m), 1595 (m) cm^{-1}. **Elemental Analysis:** found (calcd.): C 24.98 (25.61), H 2.17 % (2.15 %),

^{1}H-NMR ($CDCl_3$, 300 MHz)**:** δ = 1.01 (m, 3H, -CH_3), 1.12-1.68 (m, 33H, C_6H_{11}), 1.79 (m, 2H, -SnCH_2-), 2.80 (m, 2H, -CH_2-) 4.29 (q, $^1J_{HH}$ =7.1 Hz, 2H, -OCH_2), 7.45 (d, $^3J_{HH}$ = 7.2 Hz, 2H, CH*arom*), 7.89 (d, $^3J_{HH}$ = 8.2 Hz, 2H, CH*arom*) ppm. **^{13}C-NMR** (CDCl3, 75 MHz)**:** δ = 8.4, 14.1, 26.1, 27.0, 29.0, 32.2, 33.3, 60.4, 127.3, 127.7, 129.5, 151.6, 166.5 ppm. **^{119}Sn-NMR** ($CDCl_3$, 187 MHz)**:** δ = –66 ppm. **HRMS (ESI+):** m/z calcd: 569.2417 [$C_{29}H_{46}O_2SnNa$]$^+$, found: 569.2420.

Synthesis of [$(R^1Sn)_4S_6$] (4)

C (370 mg, 0.92 mmol) was dissolved in toluene (15 mL). $(Me_3Si)_2S$ (0.30 mL, 1.43 mmol) was added and the solution stirred at room temperature for two hours. After solvent removal *in vacuo*, the residue was taken up in *n*-pentane (10 mL), filtered and dried. **4** (219 mg, 0.16 mmol, 69%) was obtained as a light yellow solid.

^{1}H-NMR ($CDCl_3$, 300 MHz)**:** δ = 1.39 (t, $^3J_{HH}$ = 7.1 Hz, 3H, -CH_3), 2.12 (t, $^3J_{HH}$ = 7.8 Hz, 2H, -SnCH_2-), 3.16 (t, $^3J_{HH}$ = 7.8 Hz, 2H, -CH_2-), 4.37 (q, $^1J_{HH}$ = 7.1 Hz, 2H, -OCH_2), 7.30 (d, $^3J_{HH}$ = 8.1 Hz, 2H, -CH*arom*), 8.00 (d, $^3J_{HH}$ = 8.2 Hz, 2H, -CH*arom*) ppm. **^{13}C-NMR** (CDCl3, 75 MHz)**:** δ = 14.9, 30.5, 31.2, 61.5, 128.6, 129.6, 130.7, 147.6, 167.1 ppm. **^{119}Sn-NMR** ($CDCl_3$, 187 MHz)**:** δ = 141 ($^1J_{SnC}$ = 109 Hz) ppm. **HRMS (ESI+):** m/z calcd: 1396.7962 [$C_{44}H_{52}O_8Sn_4S_6Na$]$^+$, found: 1396.7970. **IR:** $\tilde{\nu}$ = 3018 (w), 2957 (w), 2921 (w), 2847 (w), 1665 (w), 1590 (m), 1489 (m), 1451 (m), 1412 (m), 1333 (w), 1248 (w), 1203 (w), 1177 (w), 1152

(w), 1091 (w), 1043 (m), 1027 (m), 901 (m), 861 (m), 840 (w), 816 (w), 795 (w), 752 (m), 722 (m), 688 (m), 619 (w),563 (w), 548 (m), 622 (w), 561 (w), 548 (m), 497 (w), 476 (w), 400 (s) cm^{-1}. **Elemental Analysis:** found (calcd.): C 37.52 (38.40), H 4.12 (3.81), S 13.24 % (13.98 %).

Synthesis of $[(R^1Sn)_4Se_6]$ (5)

C (107 mg, 0.266 mmol) was dissolved in toluene (7 mL) and reacted with $(Me_3Si)_2Se$ (0.10 mL, 0.400 mmol). The clear yellow solution was stirred at room temperature for sixteen hours. Solvent removal *in vacuo* afforded **5** (97 mg, 0.058 mmol, 88%) as a dark orange solid.

^{1}H-NMR ($CDCl_3$, 300 MHz)**:** δ = 1.37 (t, 3H, -CH_3), 2.21 (t, 2H, -$SnCH_2$-), 3.09 (t, 2H, -CH_2-), 4.34 (q, 2H, -OCH_2-), 7.34 (d, 2H, CH_{arom}), 7.98 (d, 2H, CH_{arom}) ppm. **^{13}C-NMR** (CDCl3, 75 MHz)**:** δ = 14.7, 29.2, 32.0, 61.4, 128.8, 129.6, 130.5, 147.7, 166.8 ppm. **^{119}Sn-NMR** ($CDCl_3$, 187 MHz)**:** δ = –33 ($^1J_{SnC}$ = 133 Hz, $^1J_{SnSe}$ = 749 Hz) ppm. **IR:** $\tilde{\nu}$ = 2962 (m), 2907 (m), 2840 (m), 1708 (s), 1606 (m), 1574 (w), 1507 (w), 1455 (w), 1441 (w), 1413 (m), 1389 (w), 1362 (m), 1305 (w), 1258 (s), 1173 (m), 1095 (s), 1016 (s), 791 (s), 753 (m), 703 (m), 665 (w), 634 (w), 597 (w), 571 (w), 485 (w) cm^{-1}. **Elemental Analysis:** found (calcd.): C 32.25 (31.88), H 3.24 % (3.16 %).

Synthesis of $[(CpSn)_4S_6]$ (6)

NaCp (754 mg, 8.56 mmol) was suspended in toluene (20 mL), cooled to 0°C and a solution of $SnCl_4$ (1.00 mL, 8.56 mmol) in toluene (10 mL) was added dropwise. After stirring for 5 hours, the reaction mixture was filtered to remove NaCl. $(Me_3Si)_2S$ (2.70 mL, 12.86 mmol) was added to the filtrate. After ca. 10 min, a light yellow precipitate formed. Stirring was continued for one hour and the product collected by filtration and drying *in vacuo*. **6** (1.15 g, 1.24 mmol, 58%) was obtained as a yellow solid.

^{1}H-NMR (CD_2Cl_2, 300 MHz)**:** δ = 6.24 (s, 20H, C_5H_5) ppm. **^{13}C-NMR** (CD_2Cl_2, 75 MHz)**:** δ = 116.9 (s, C_5H_5) ppm. **^{119}Sn-NMR** (CD_2Cl_2, 112 MHz)**:** δ = 145 ($^1J_{SnC}$ = 100Hz) ppm **IR:** $\tilde{\nu}$ = 1380 (w), 1288 (w), 1250 (w), 1108 (w), 1085 (w), 1019 (w), 981 (w), 953 (w), 919 (m), 897 (m), 815 (w), 745 (s), 638 (m), 562 (w), 464 (w) cm^{-1}. **Elemental Analysis:** found (calcd.): C 21.91 (25.90), H 1.99 (2.17), S 21.08 % (20.74 %).

Synthesis of $[(CpSn)_4Se_6]$ (7)

NaCp (754 mg, 8.56 mmol) was suspended in toluene (20 mL), cooled to 0°C and a solution of $SnCl_4$ (1.00 mL, 8.56 mmol) in toluene (10 mL) was added dropwise. After stirring for 5

hours, the reaction mixture was filtered to remove NaCl. $(Me_3Si)_2Se$ (3.30 mL, 13.2 mmol) was added to the filtrate. Immediately, an orange precipitate was formed that was collected by filtration and dried *in vacuo*. **7** (1.22 g, 1.01 mmol, 47%) was obtained as an orange powder.

^{1}H-NMR (CD_2Cl_2, 300 MHz)**:** δ = 6.27 (s, 20H, C_5H_5) ppm. **^{13}C-NMR** (CD_2Cl_2, 75 MHz)**:** δ = 116.7 (s, C_5H_5) ppm. **^{119}Sn-NMR** (CD_2Cl_2, 112 MHz)**:** δ = –113 ($^1J_{SnC}$ = 153 Hz, $^1J_{SnSe}$ = 764 Hz) ppm. **IR:** $\tilde{\nu}$ = 1430 (w), 1377 (w), 1288 (w), 1223 (w), 1007 (w), 1083 (w), 1020 (w), 982 (w), 952 (w), 892 (m), 815 (w), 741 (s), 630 (m), 565 (w), 463 (w) cm^{-1}. **Elemental Analysis:** found (calcd.): C 19.73 (19.87), H 1.71 % (1.67 %).

Synthesis of $CySnCl_3$ (D)

Tetracyclohexyltin (6.93 g, 15.4 mmol) and $SnCl_4$ (5.40 mL, 46.2 mmol) were combined and heated to 140°C for 3 hours. During this time, the initially yellow suspension turned grey. Fractional distillation afforded **D** (7.40 g, 24.0 mmol, 39%) as a colorless oil.

Boiling point: 36°C/1.2·10^{-2} mbar. **^{1}H-NMR** ($CDCl_3$, 300 MHz)**:** δ = 0.99 (t, $^3J_{HH}$ = 7.3 Hz, 4H), 1.51 (m, 3H), 1.91 (dt, $^3J_{HH}$ = 12.6 , 7.5 Hz, 2H), 2.39 (t, $^3J_{HH}$ = 7.5 Hz, 2H) ppm.

Synthesis of $[(CySn)_4S_6]$ (8)

D (0.50 mL, 3.24 mmol) was dissolved in toluene (20 mL), $(Me_3Si)_2S$ (1.05 mL, 5.00 mmol) was added and the clear colorless solution stirred at room temperature for one day. Removal of all volatile components *in vacuo* afforded **8** (0.79 g, 0.79 mmol, 98%) as a colorless solid.

^{1}H-NMR (CD_2Cl_2, 300 MHz)**:** δ = 0.94 (m, 16H), 1.45 (m, 12H), 1.80 (m, 16H) ppm. **^{13}C-NMR** (CD_2Cl_2, 75 MHz)**:** δ = 13.9, 26.5, 27.6, 30.5 ppm. **^{119}Sn-NMR** (CD_2Cl_2, 112 MHz)**:** δ = 145 ($^1J_{SnC}$ = 100 Hz) ppm. **IR:** $\tilde{\nu}$ = 2954 (m), 2913 (m), 2850 (m), 1455 (m), 1375 (m), 1337 (m), 1287 (w), 1241 (m), 1171 (m), 1142 (m), 1072 (w), 1014 (m), 958 (m), 865 (w), 835 (m), 766 (w), 744 (w), 701 (m), 669 (s), 630 (w), 595 (w), 509 (w), 451 (w), 407 (w) cm^{-1}. **Elemental Analysis:** found (calcd.): C 28.41 (28.83), H 4.34 (4.44), S 19.41 % (19.24 %).

Synthesis of $[(CySn)_4Se_6]$ (9)

D (0.50 mL, 3.24 mmol) was dissolved in toluene (20 mL), $(Me_3Si)_2Se$ (1.25 mL, 5.00 mmol) was added and the clear yellow solution stirred at room temperature. After 30 minutes, a light yellow precipitate was formed that was collected by filtration after an additional 30 min. Drying *in vacuo* afforded **9** (0.58 g, 0.45 mmol, 56%) as a light yellow solid.

^{1}H-NMR (CD_2Cl_2, 300 MHz)**:** δ = 0.94 (t, 4H),1.46 (m, 3H), 1.75 (m, 2H), 1.94 (t, 2H) ppm. **^{13}C-NMR** (CD_2Cl_2, 75 MHz)**:** δ = 13.7 (Sn*C*), 26.2, 28.3, 29.4 ppm. **^{119}Sn-NMR** (CD_2Cl_2, 187 MHz)**:** δ = –22 ($^{1}J_{SnC}$ = 123 Hz, $^{1}J_{SnSe}$ = 752 Hz) ppm. **IR:** $\tilde{\nu}$ = 2948 (m), 2910 (m), 2842 (m), 1452 (m), 1440 (w), 1372 (w), 1339 (w), 1289 (w), 1238 (m), 1160 (w), 1132 (m), 1069 (w), 1008 (w), 955 (w), 860 (w), 838 (m), 766 (w), 741 (w), 699 (w), 663 (s), 585 (w)m 491 (w), 409 (w) cm^{-1}. **Elemental Analysis:** found (calcd.): C 22.17 (22.50), H 3.40 % (3.46 %).

2. NMR Spectra

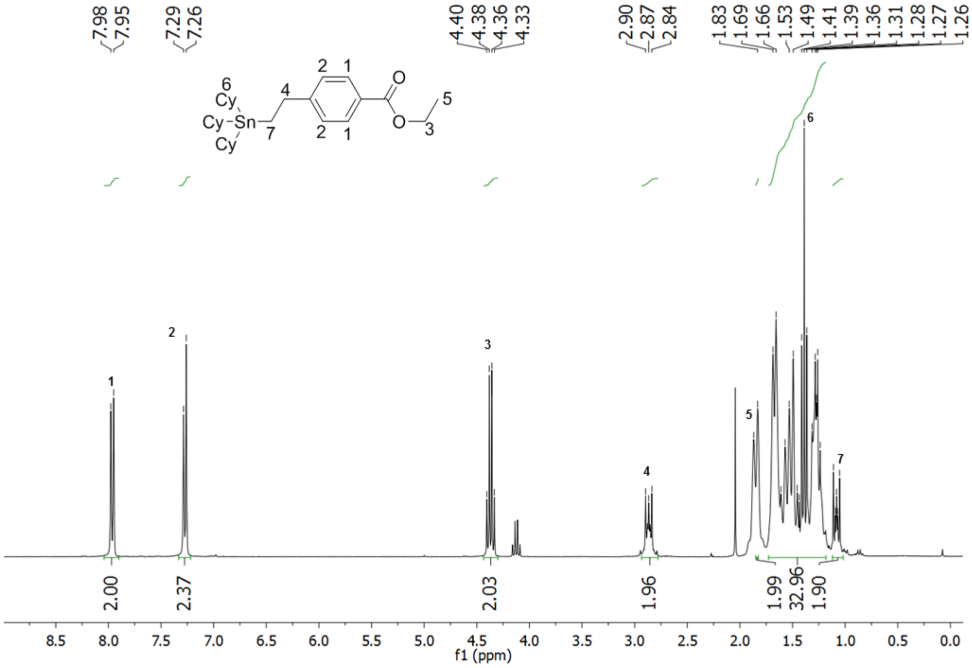

Figure S1: ^{1}H NMR spectrum of **B** (300 MHZ, 25°C, $CDCl_3$).

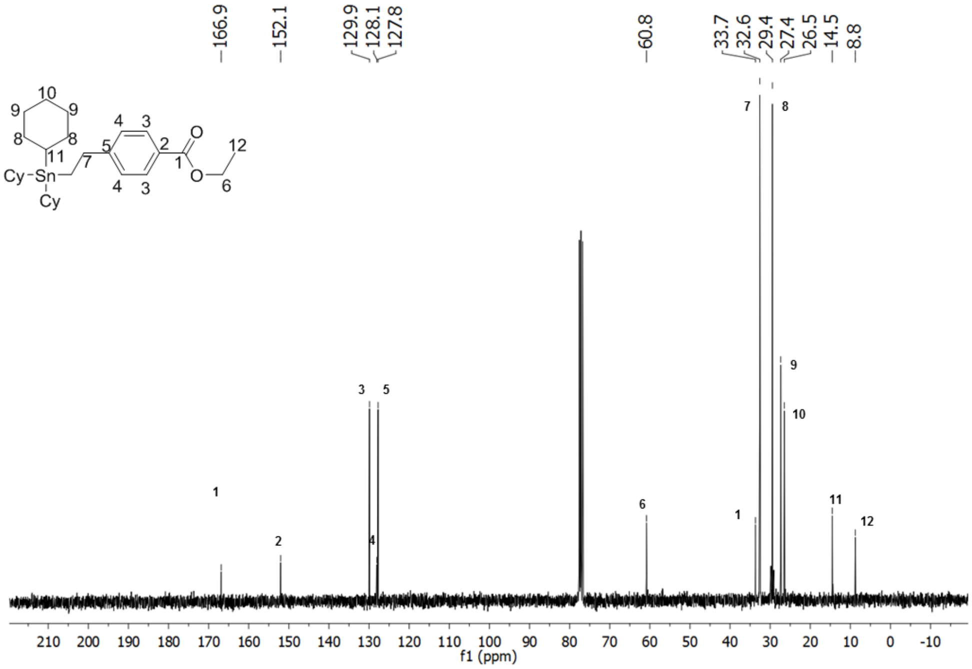

Figure S2: ^{13}C NMR spectrum of **B** (75 MHZ, 25°C, $CDCl_3$).

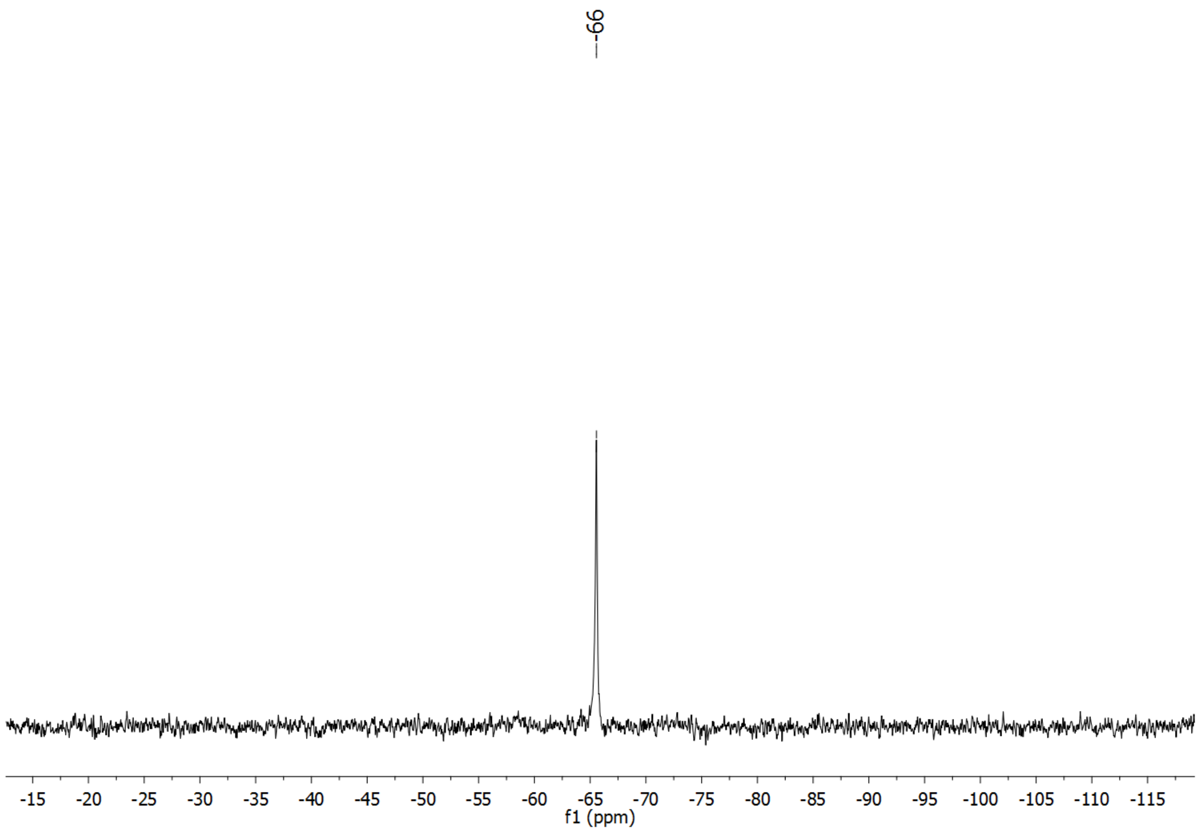

Figure S3: ^{119}Sn NMR spectrum of **B** (112 MHZ, 25°C, $CDCl_3$).

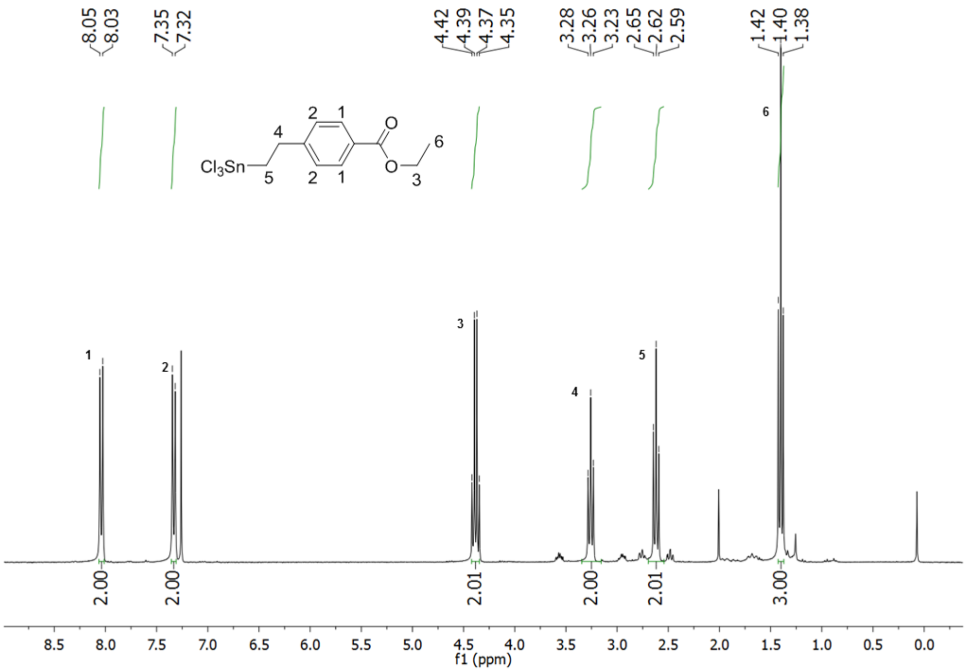

Figure S4: ^{1}H NMR spectrum of **C** (300 MHZ, 25°C, $CDCl_3$).

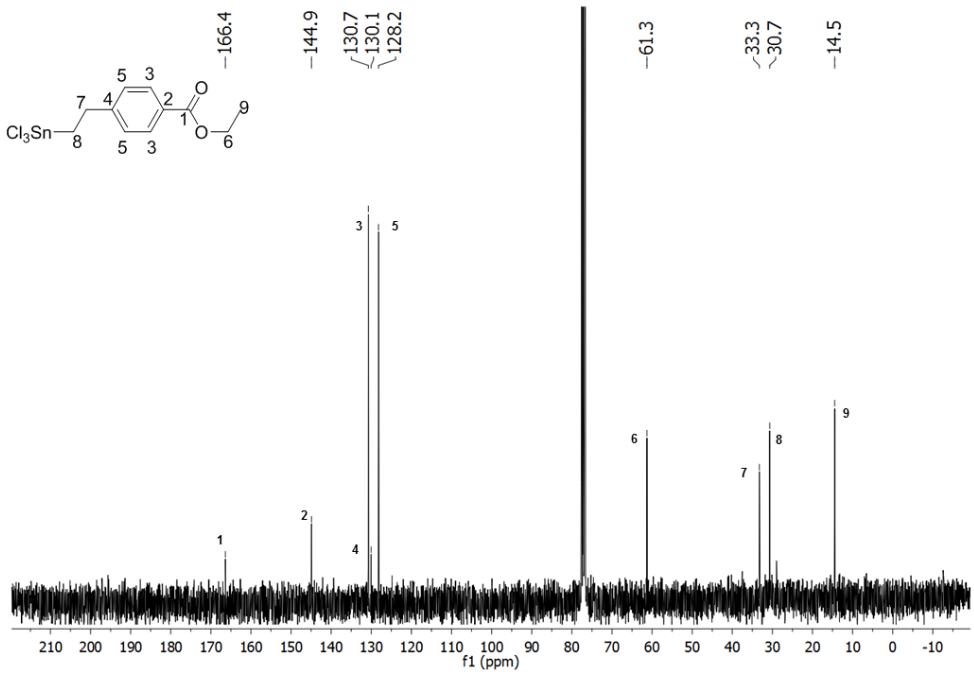

Figure S5: ^{13}C NMR spectrum of **C** (75 MHZ, 25°C, $CDCl_3$).

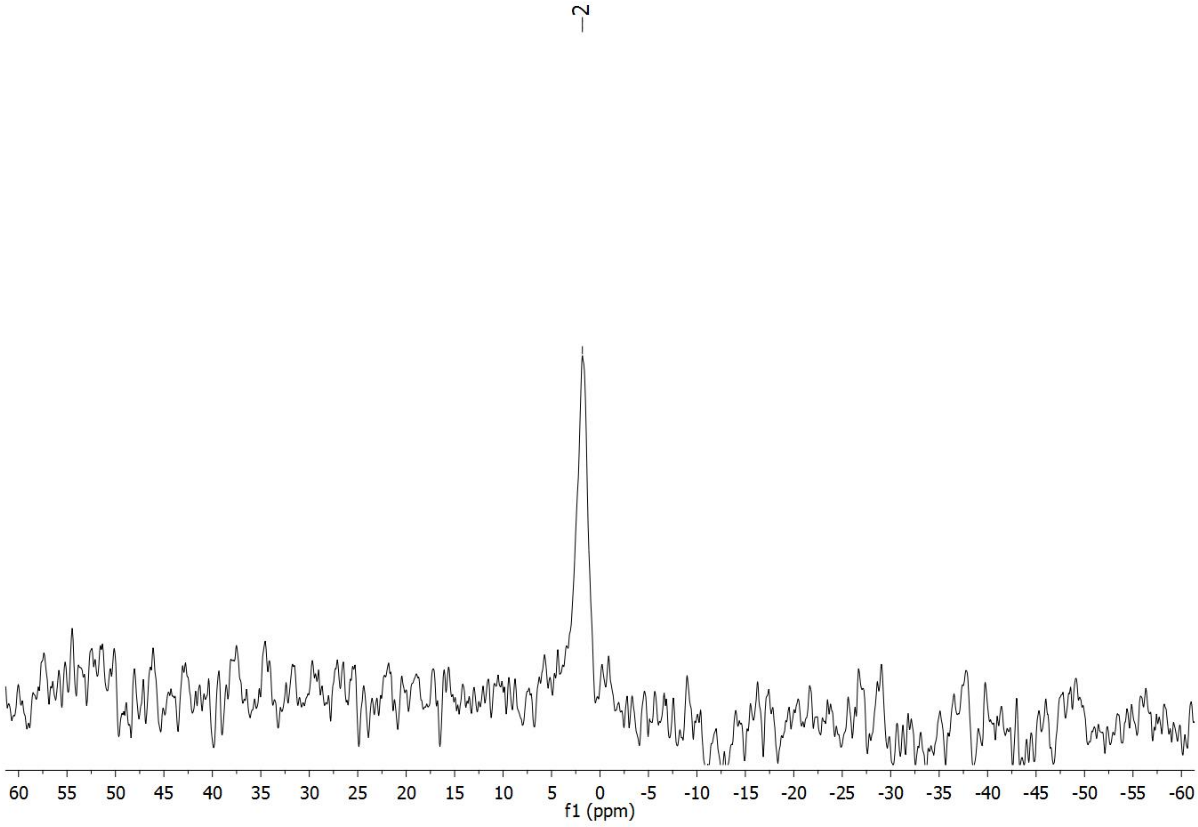

Figure S6: ^{119}Sn NMR spectrum of **C** (112 MHZ, 25°C, $CDCl_3$).

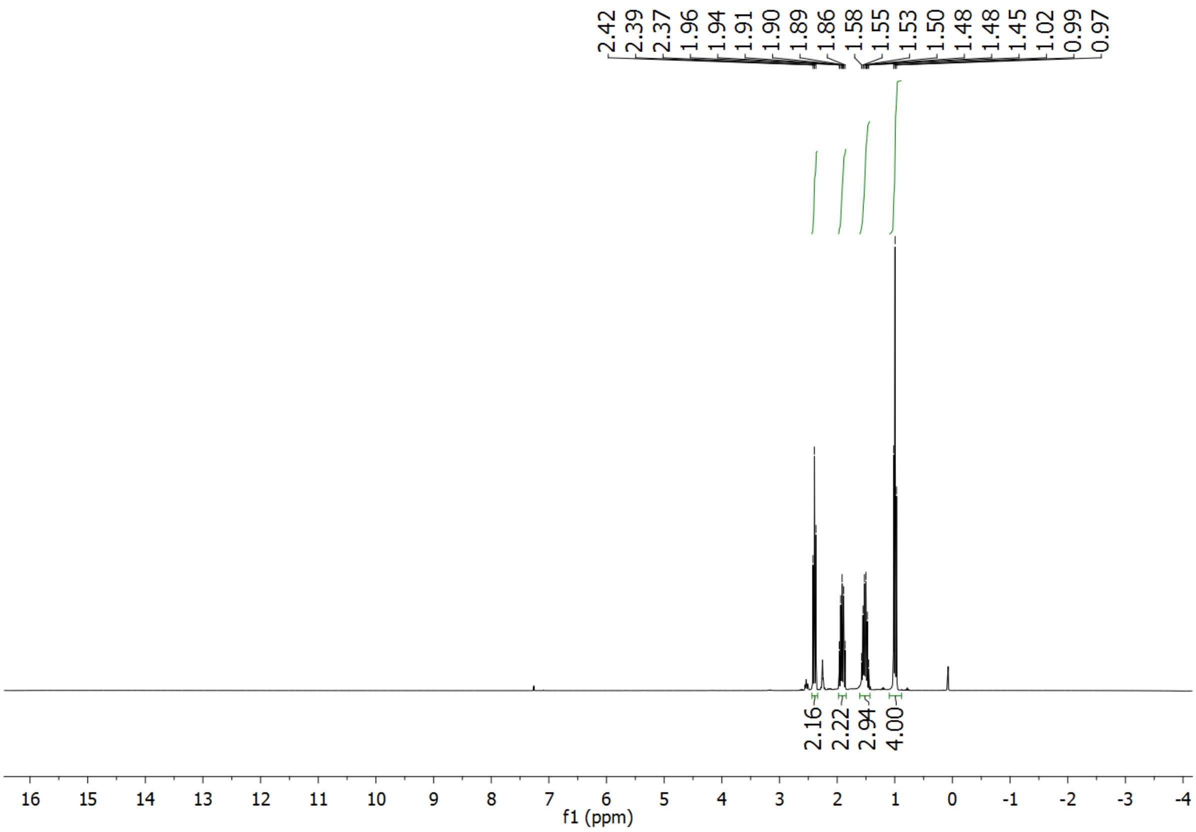

Figure S7: ^{1}H NMR spectrum of **D** (300 MHZ, 25°C, $CDCl_3$).

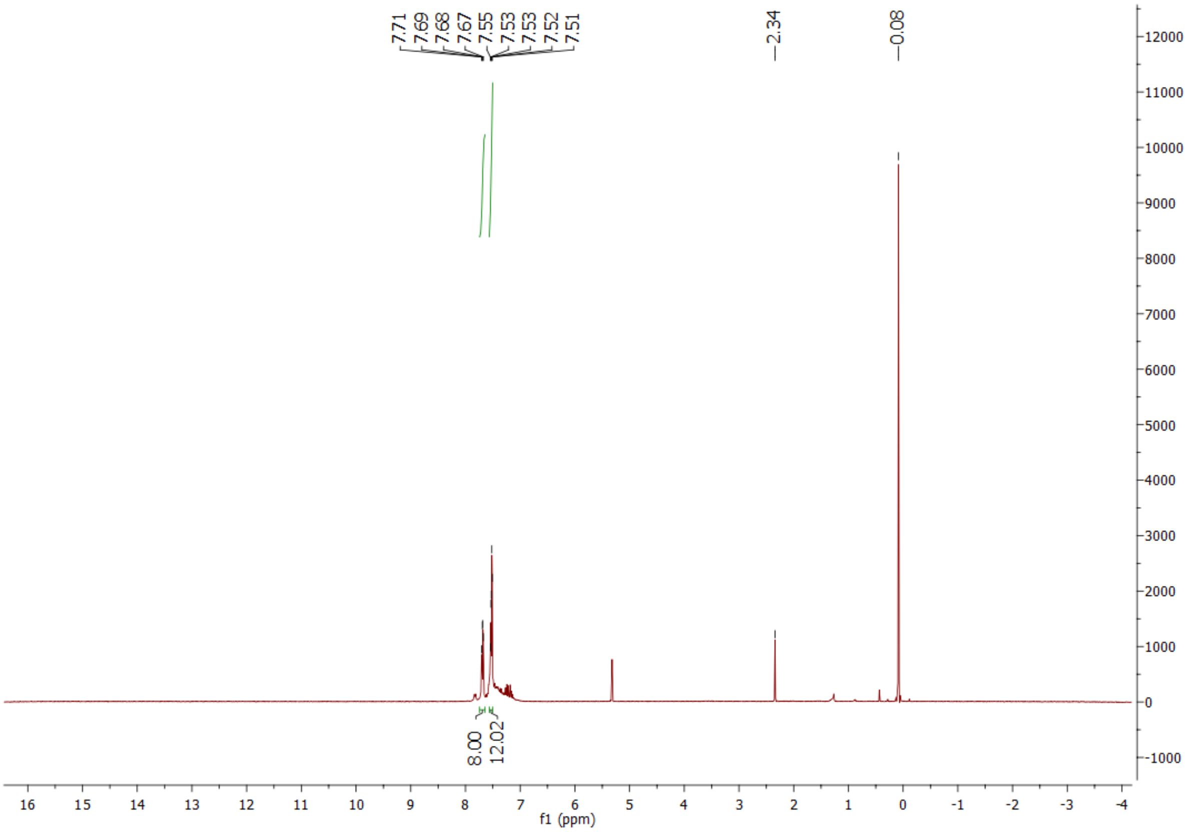

Figure S8: ^{1}H NMR spectrum of **1** (300 MHZ, 25°C, CD_2Cl_2).

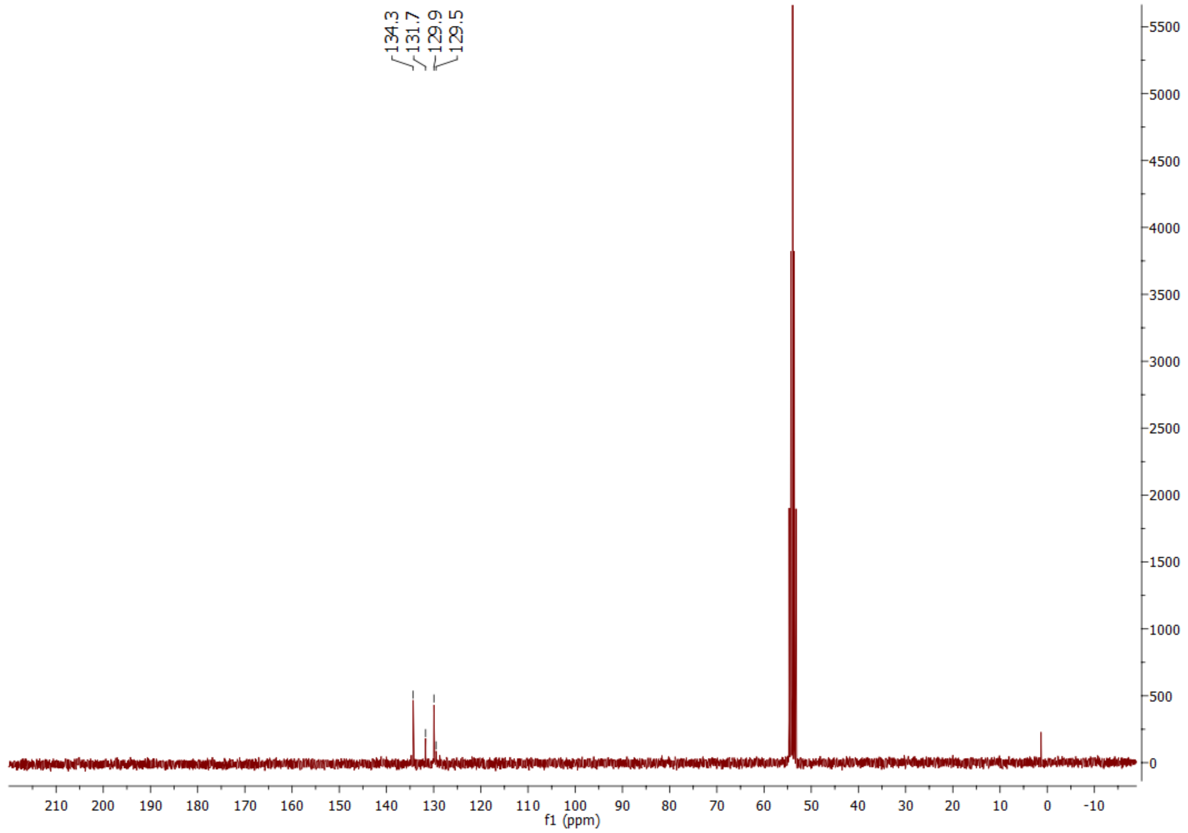

Figure S9: ^{13}C NMR spectrum of **1** (75 MHZ, 25°C, CD_2Cl_2).

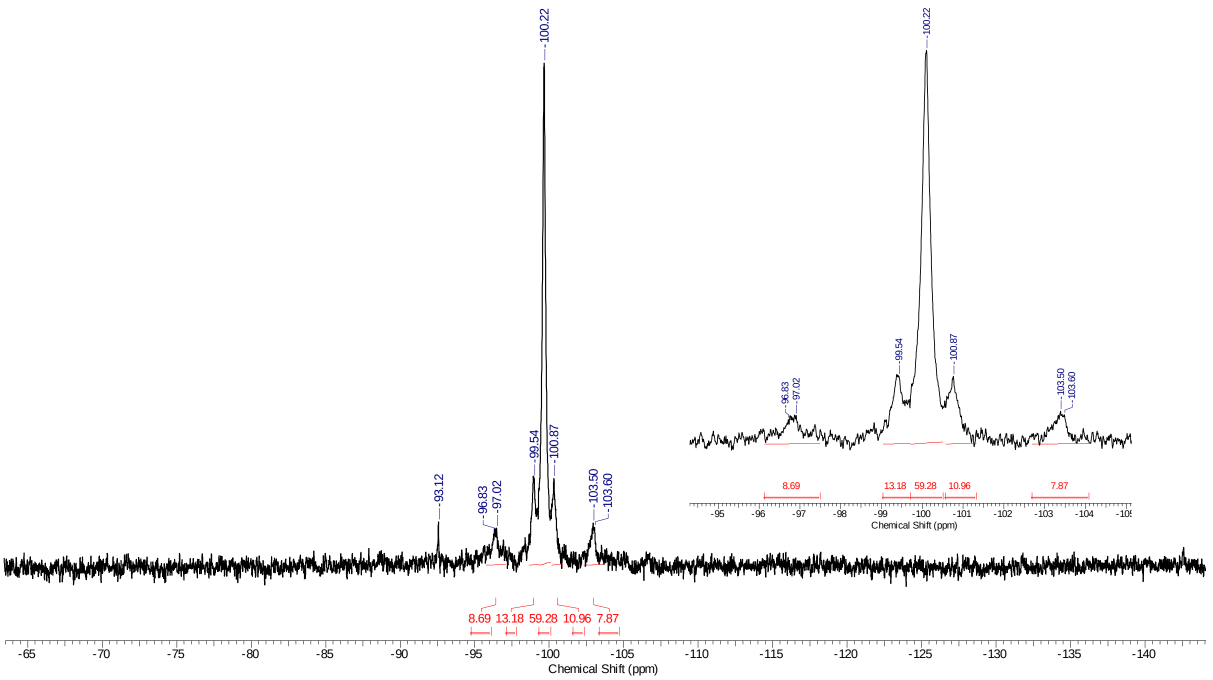

Figure S10: ^{119}Sn NMR spectrum of **1** (196 MHZ, 25°C, CD_2Cl_2).

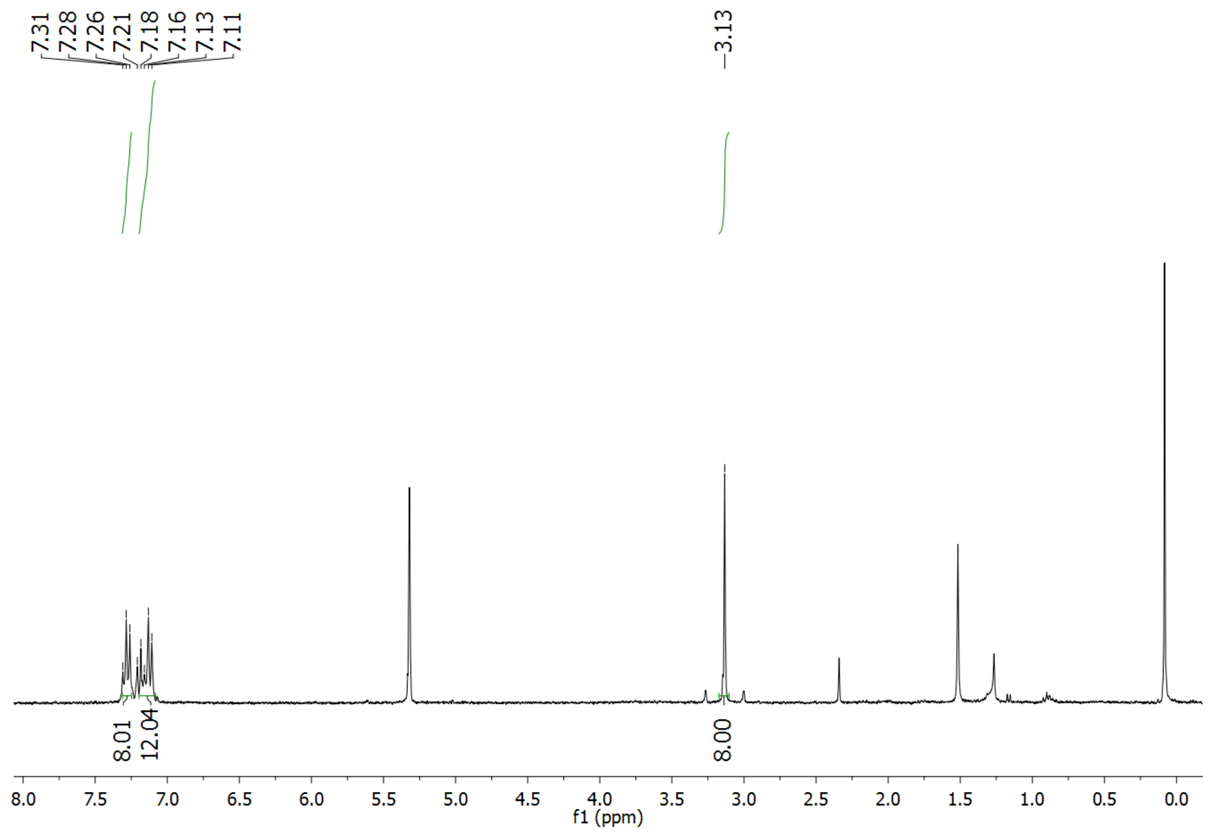

Figure S11: ^{1}H NMR spectrum of **2** (300 MHZ, 25°C, CD_2Cl_2).

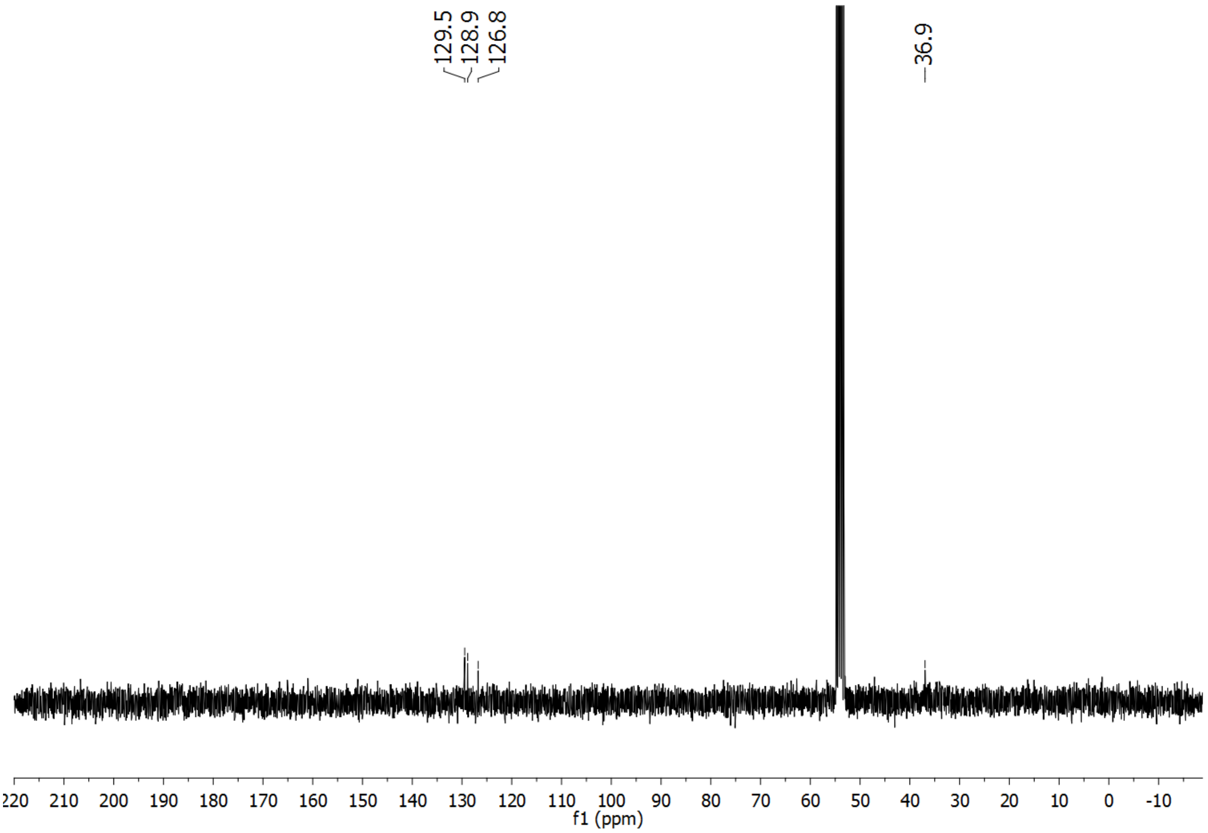

Figure S12: ^{13}C NMR spectrum of **2** (75 MHZ, 25°C, CD_2Cl_2).

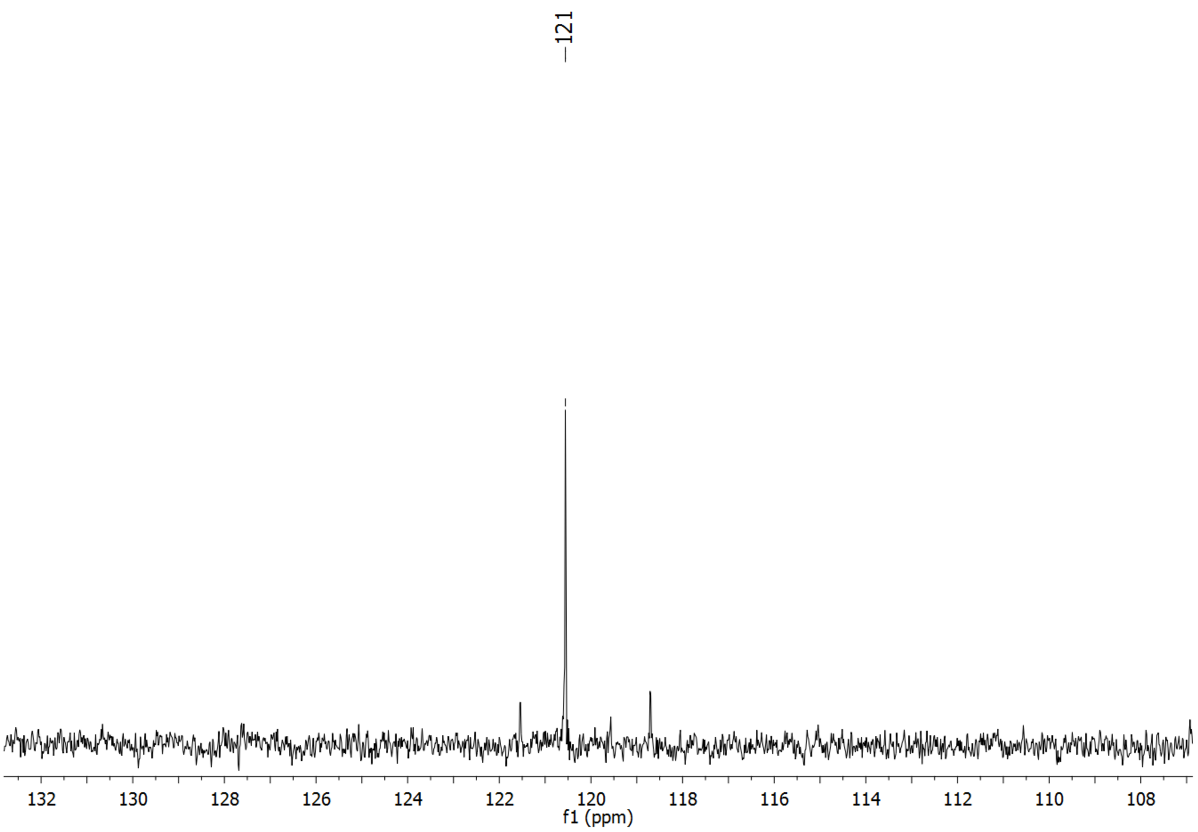

Figure S13: ^{119}Sn NMR spectrum of **2** (112 MHZ, 25°C, CD_2Cl_2).

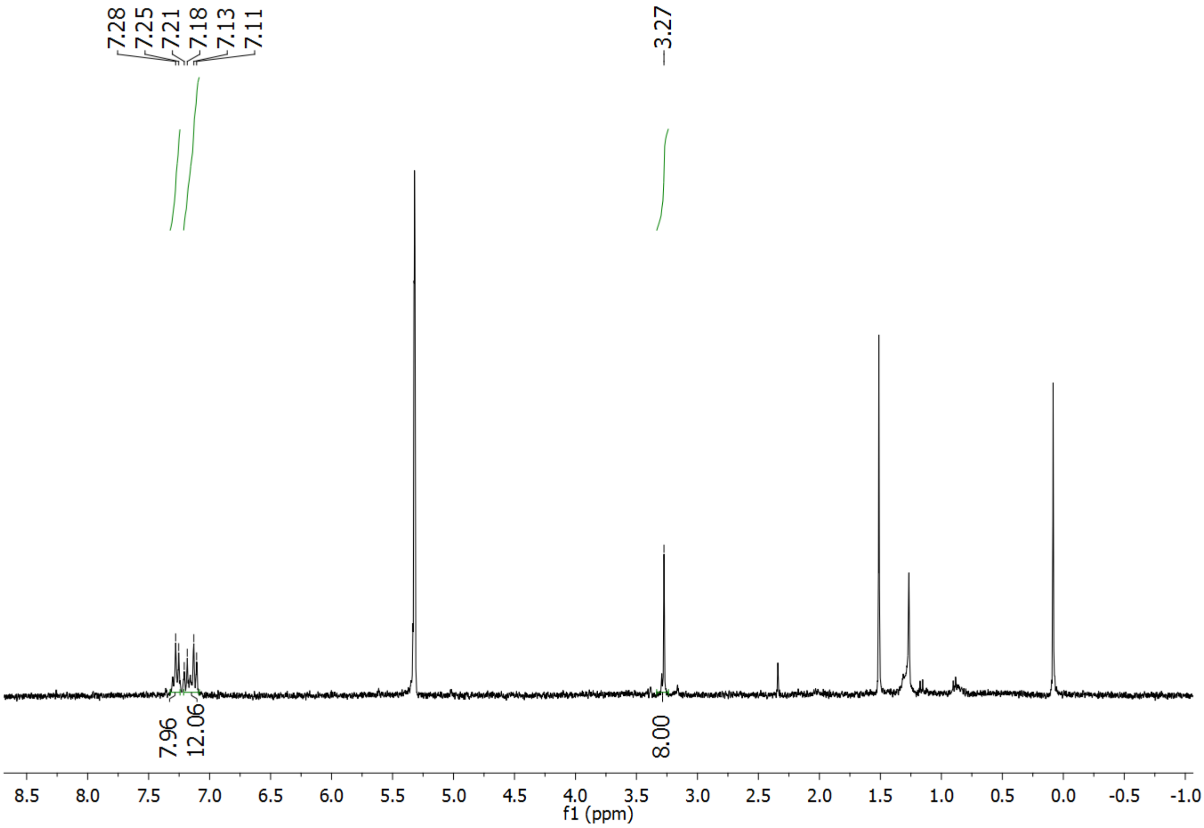

Figure S14: ^{1}H NMR spectrum of **3** (300 MHZ, 25°C, CD_2Cl_2).

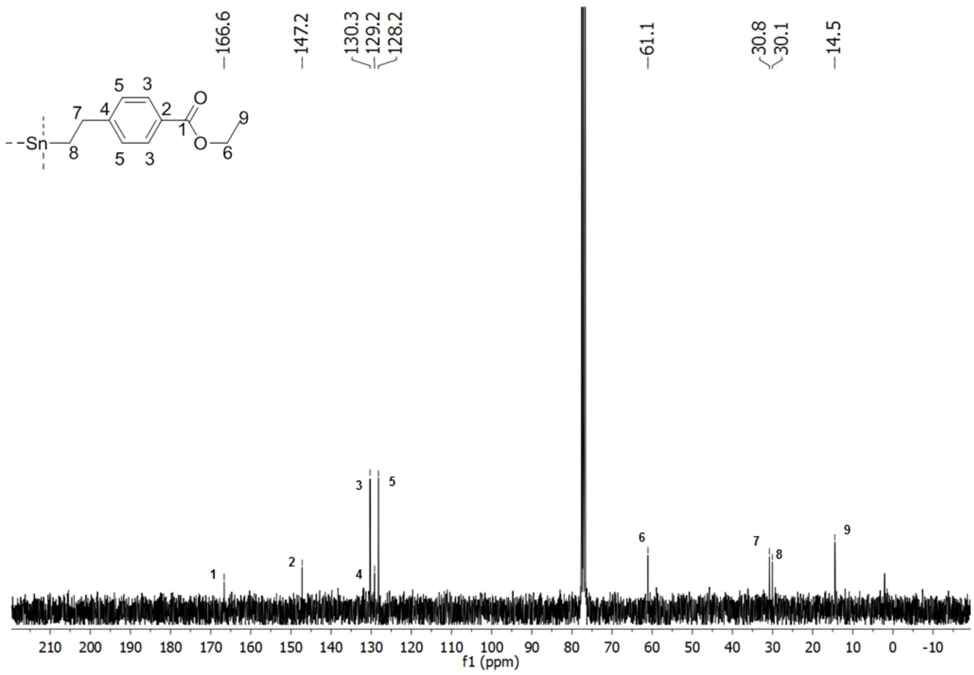

Figure S15: ^{1}H NMR spectrum of **4** (300 MHZ, 25°C, $CDCl_3$).

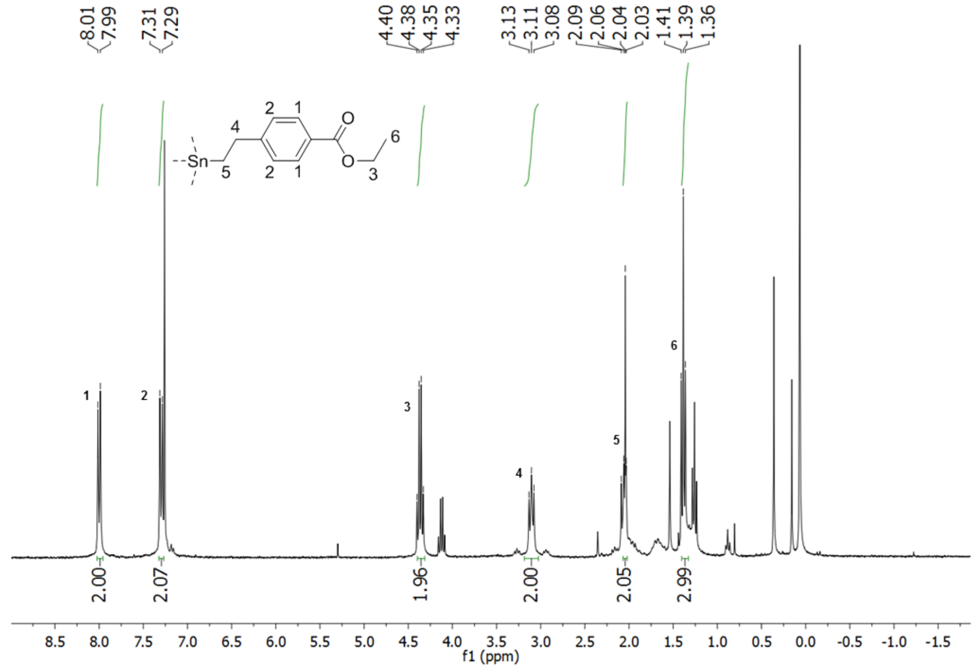

Figure S16: ^{13}C NMR spectrum of **4** (75 MHZ, 25°C, $CDCl_3$).

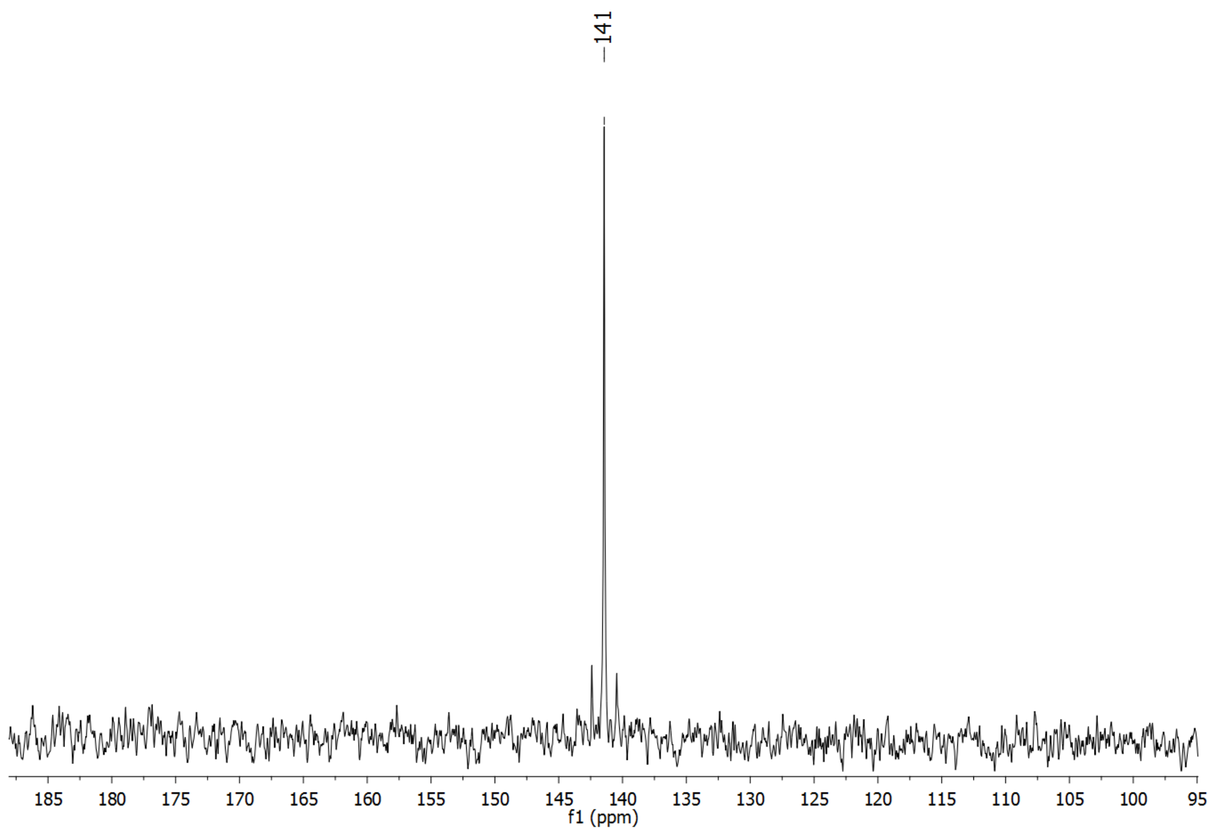

Figure S17: ^{119}Sn NMR spectrum of **4** (112 MHZ, 25°C, $CDCl_3$).

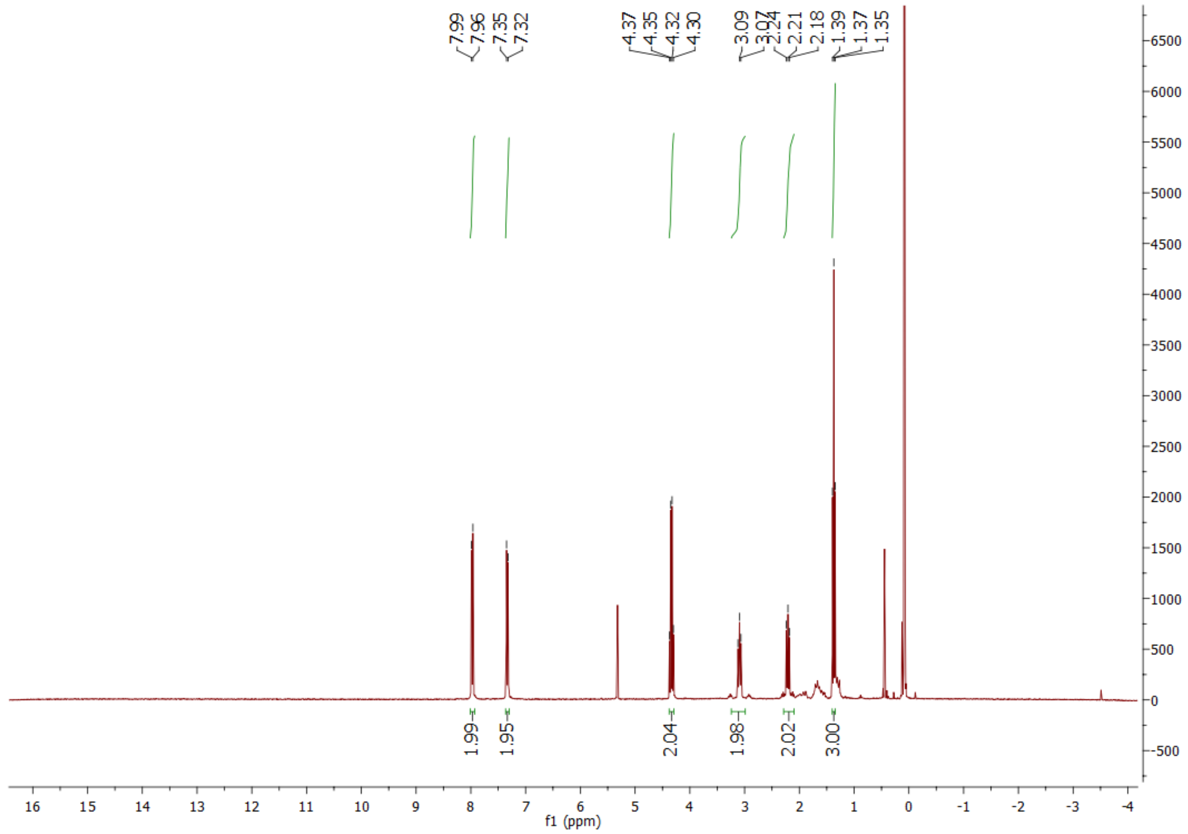

Figure S18: ^{1}H NMR spectrum of **5** (300 MHZ, 25°C, CD_2Cl_2).

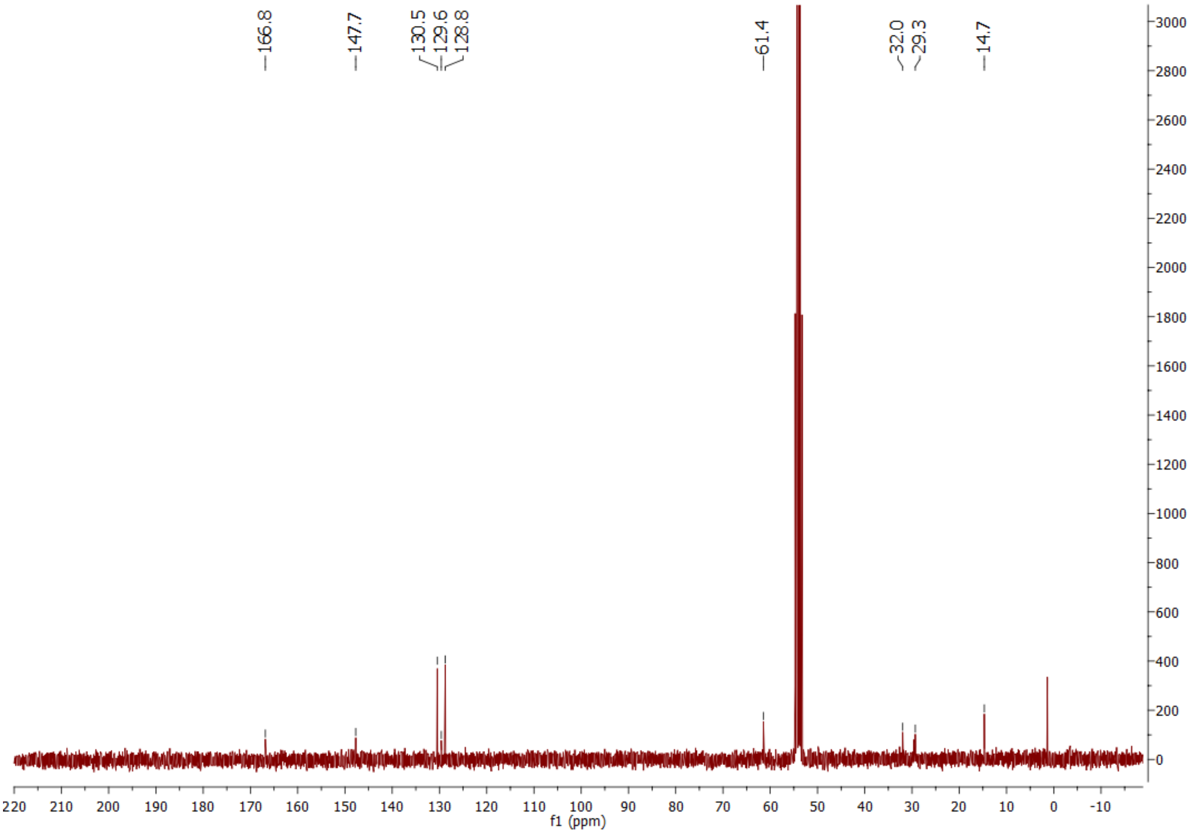

Figure S19: ^{13}C NMR spectrum of **5** (75 MHZ, 25°C, CD_2Cl_2).

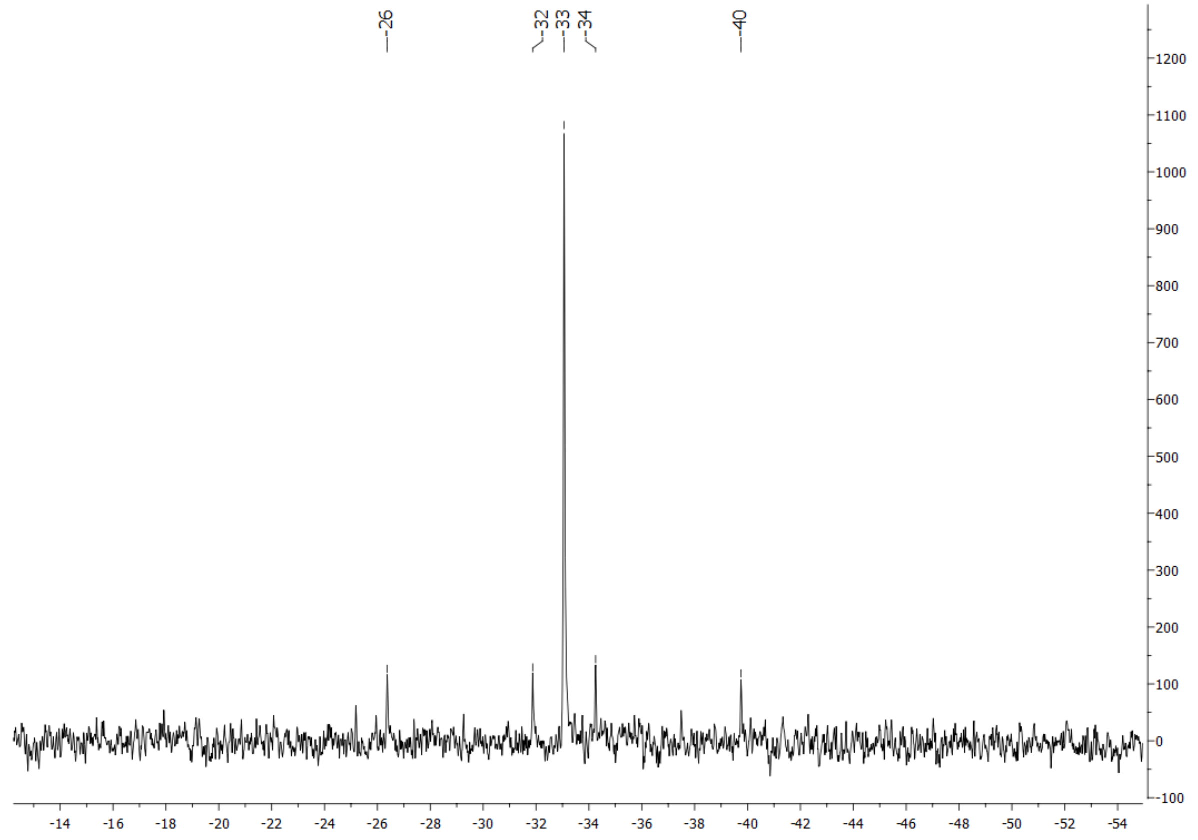

Figure S20: ^{119}Sn NMR spectrum of **5** (112 MHZ, 25°C, CD_2Cl_2).

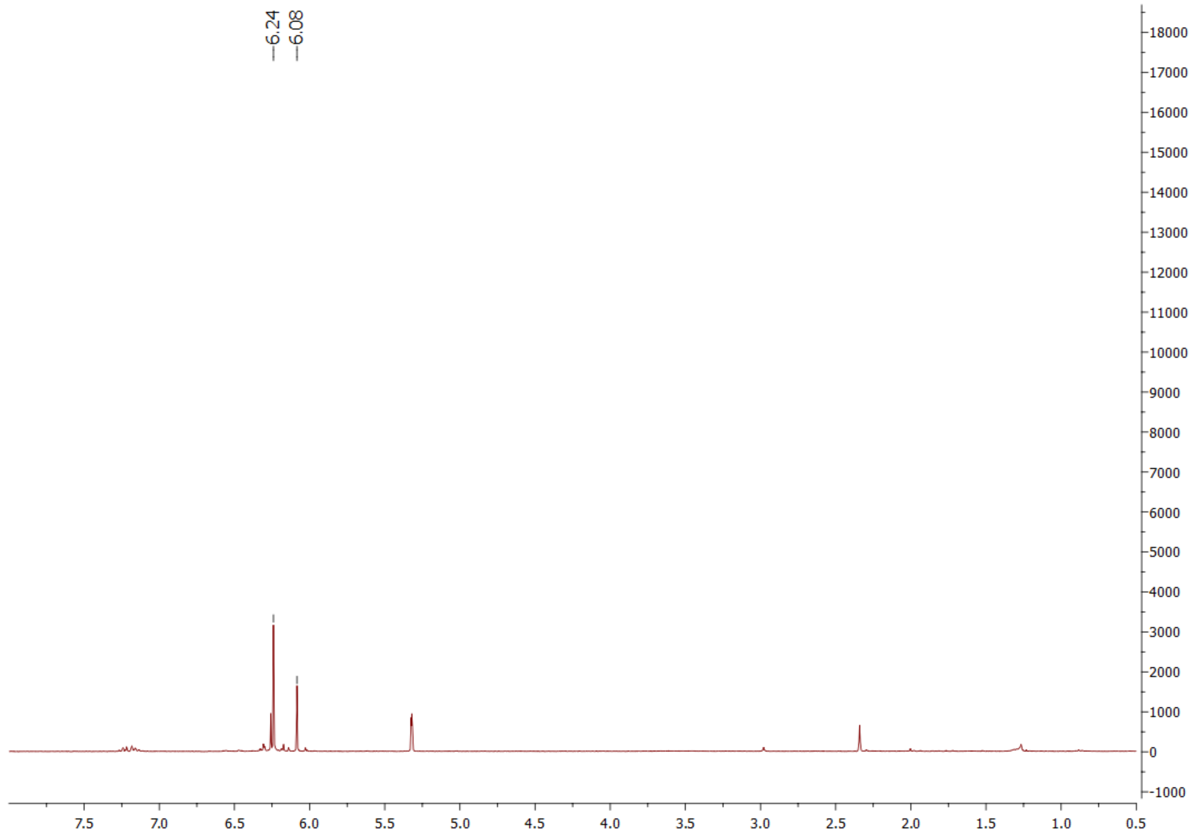

Figure S21: ^{1}H NMR spectrum of **6** (300 MHZ, 25°C, CD_2Cl_2)

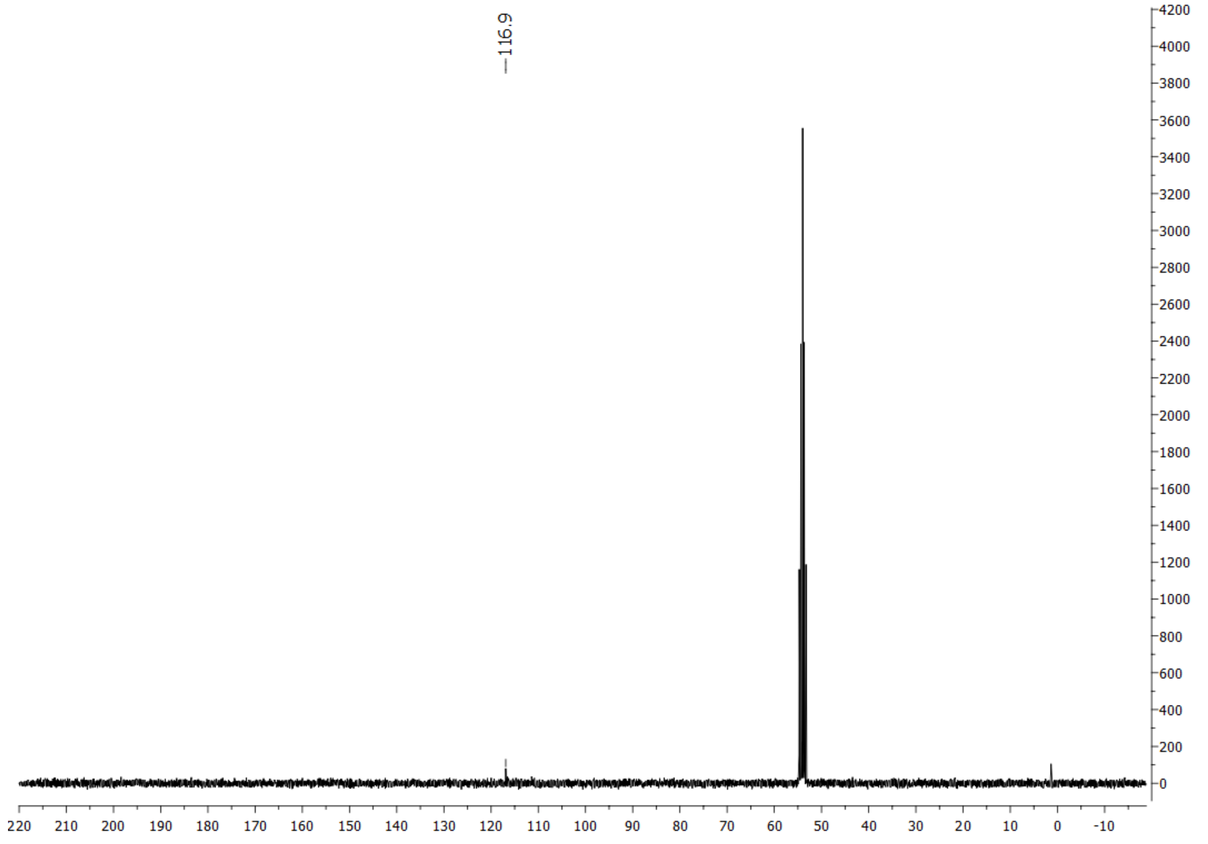

. **Figure S22:** ^{13}C NMR spectrum of **6** (75 MHZ, 25°C, CD_2Cl_2)

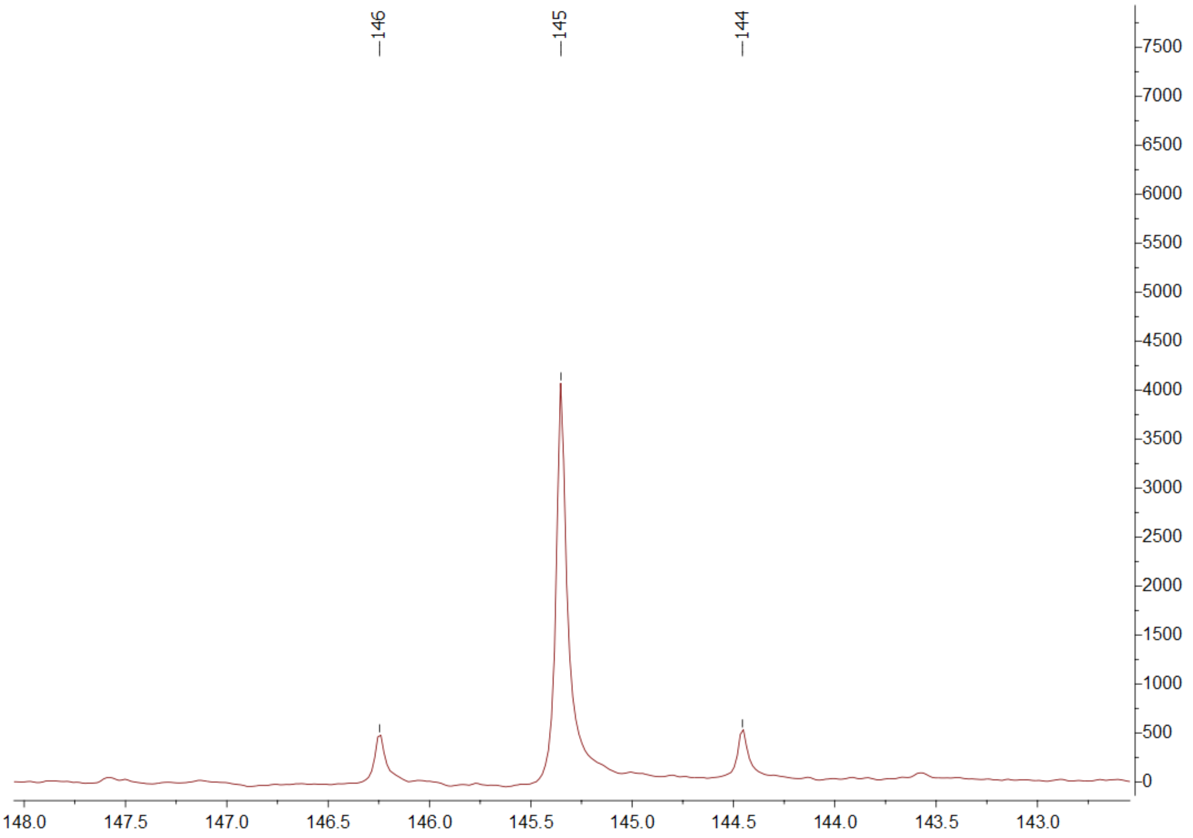

Figure S23: ^{119}Sn NMR spectrum of **6** (112 MHZ, 25°C, CD_2Cl_2)

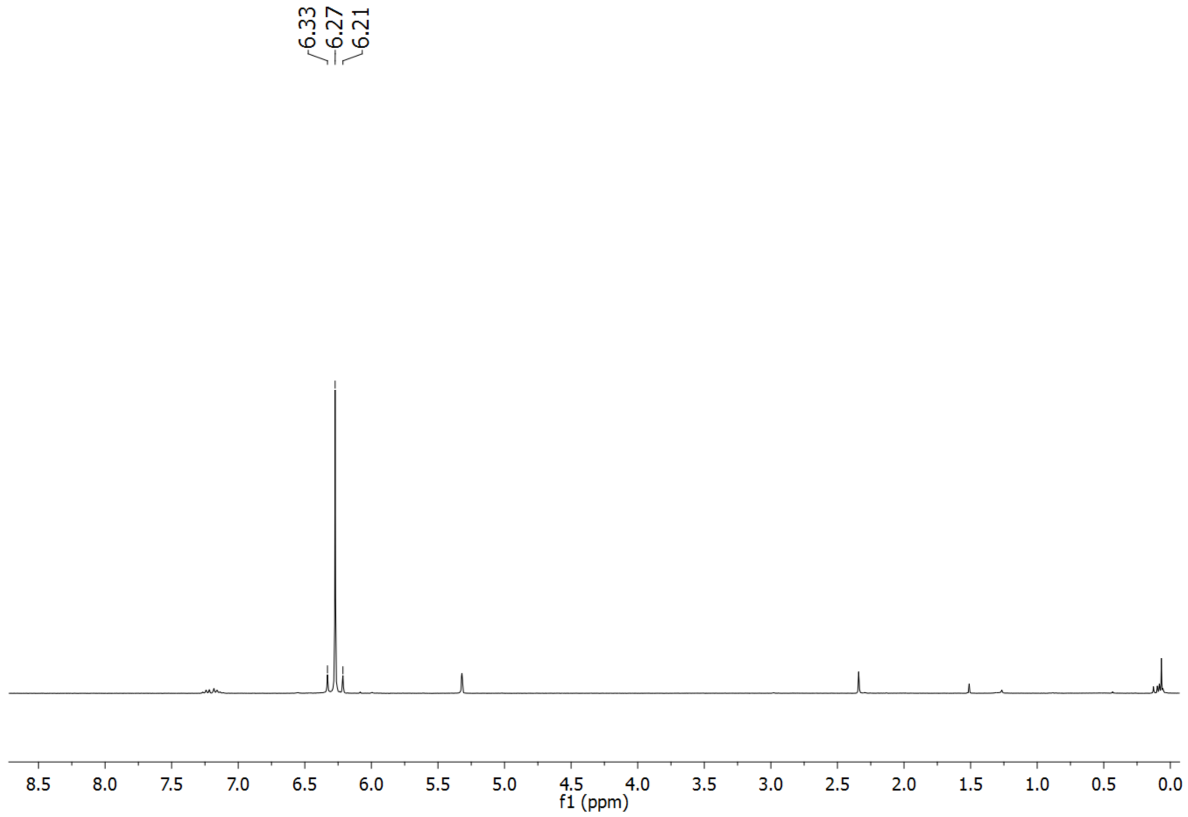

Figure S24: ^{1}H NMR spectrum of **7** (300 MHZ, 25°C, CD_2Cl_2).

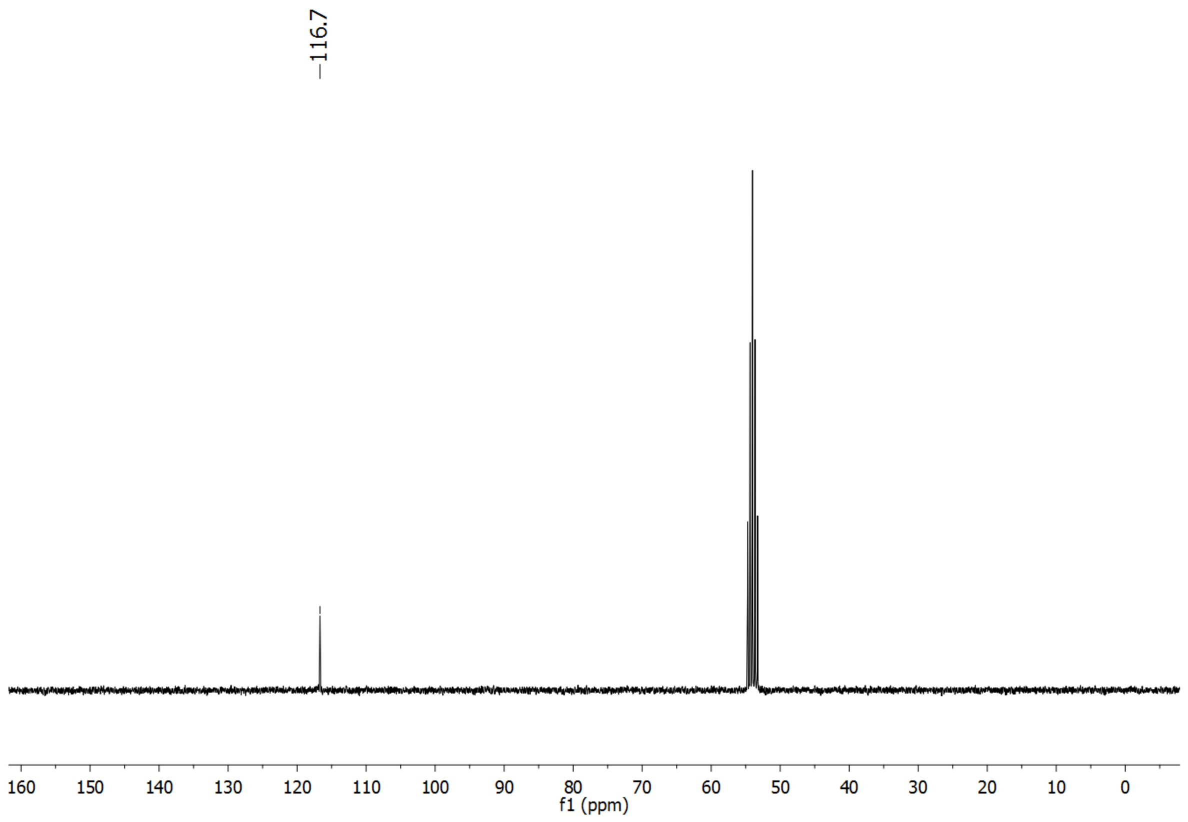

Figure S25: ^{13}C NMR spectrum of **7** (75 MHZ, 25°C, CD_2Cl_2).

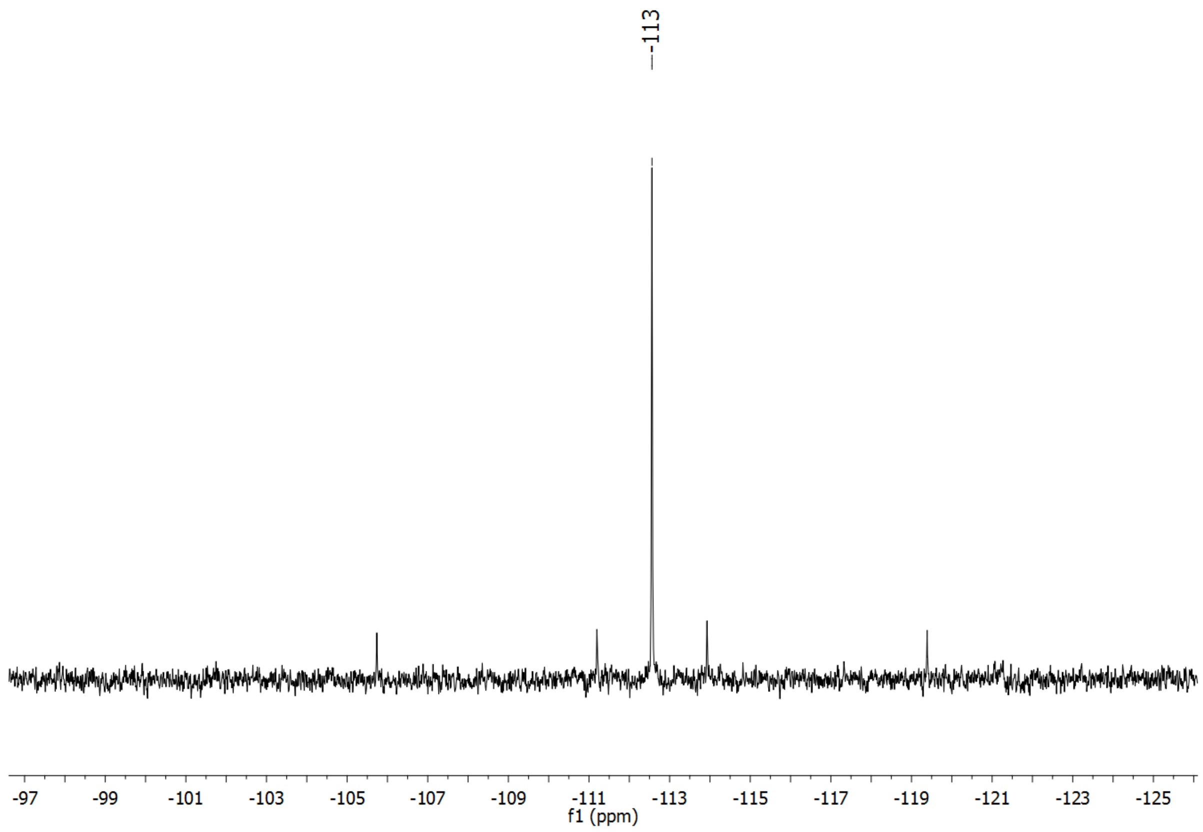

Figure S26: ^{119}Sn NMR spectrum of **7** (112 MHZ, 25°C, CD_2Cl_2).

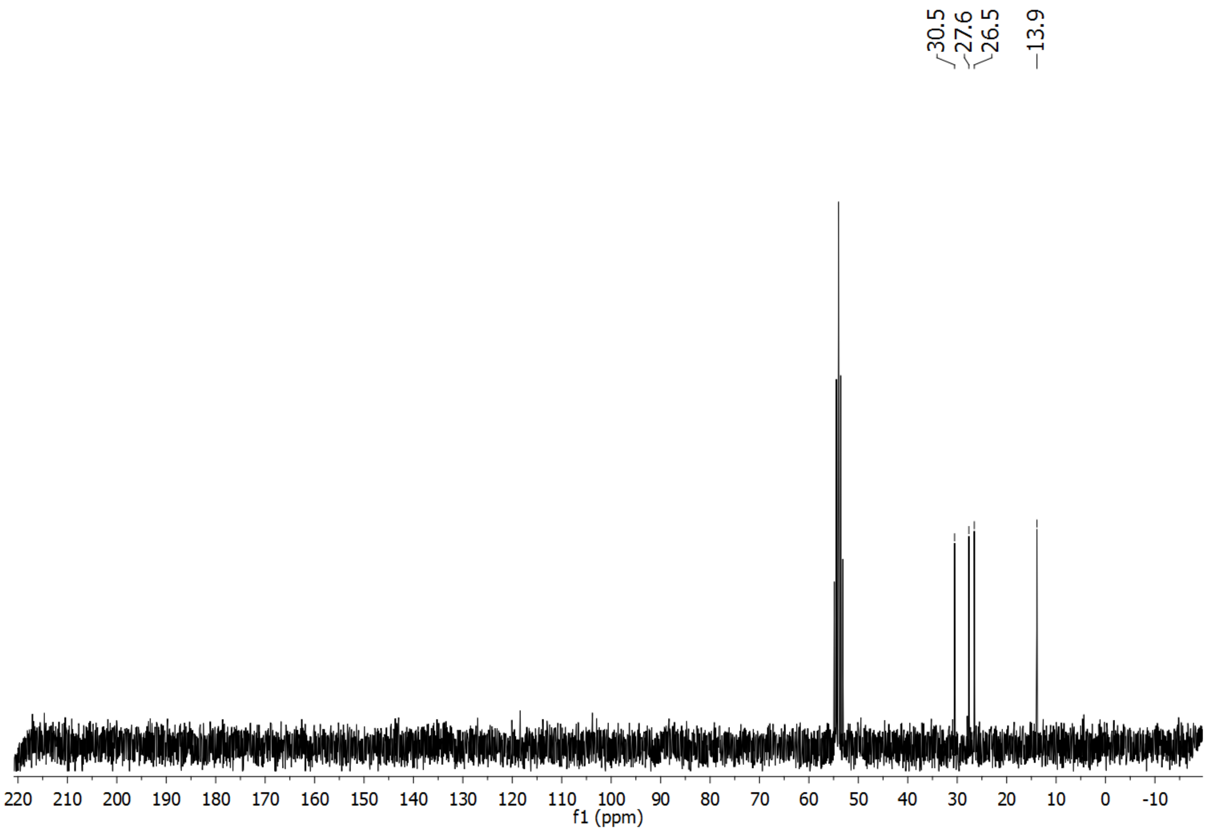

Figure S27: ^{1}H NMR spectrum of **8** (300 MHZ, 25°C, CD_2Cl_2).

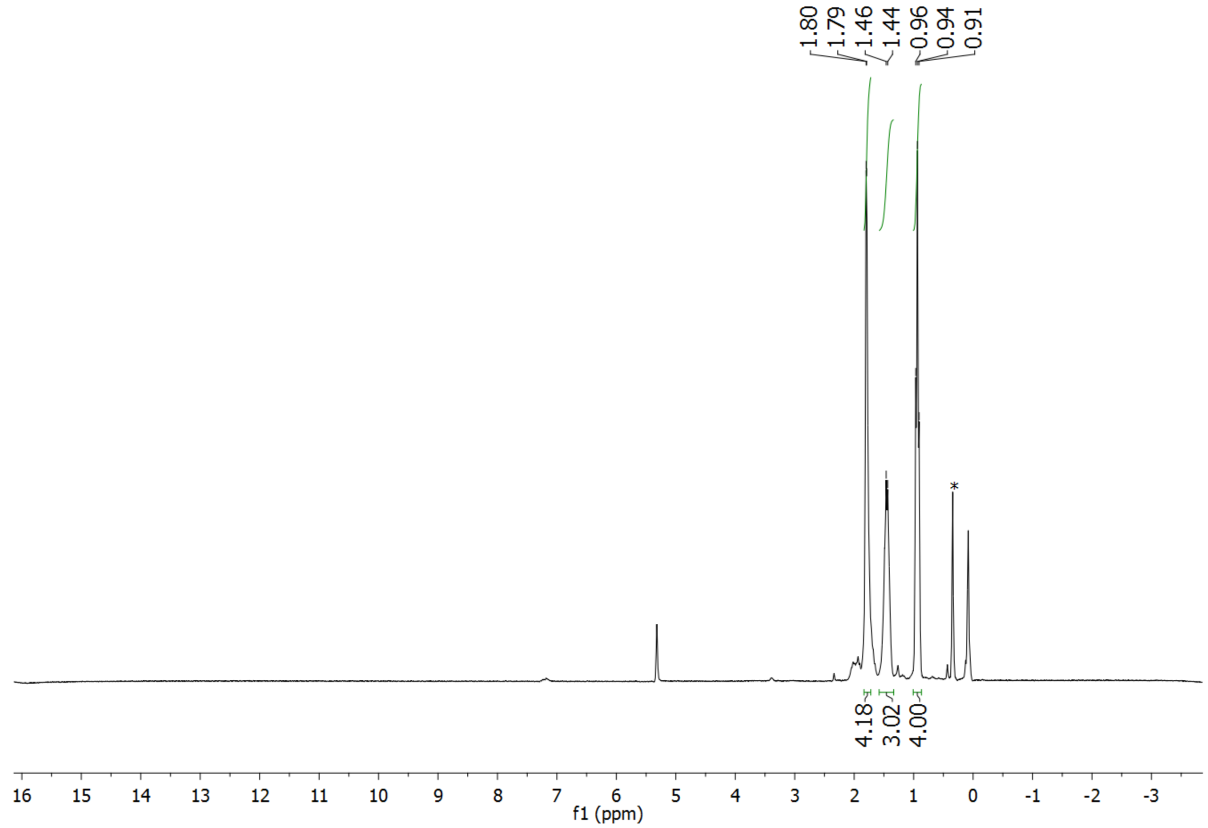

Figure S28: ^{13}C NMR spectrum of **8** (75 MHZ, 25°C, CD_2Cl_2).

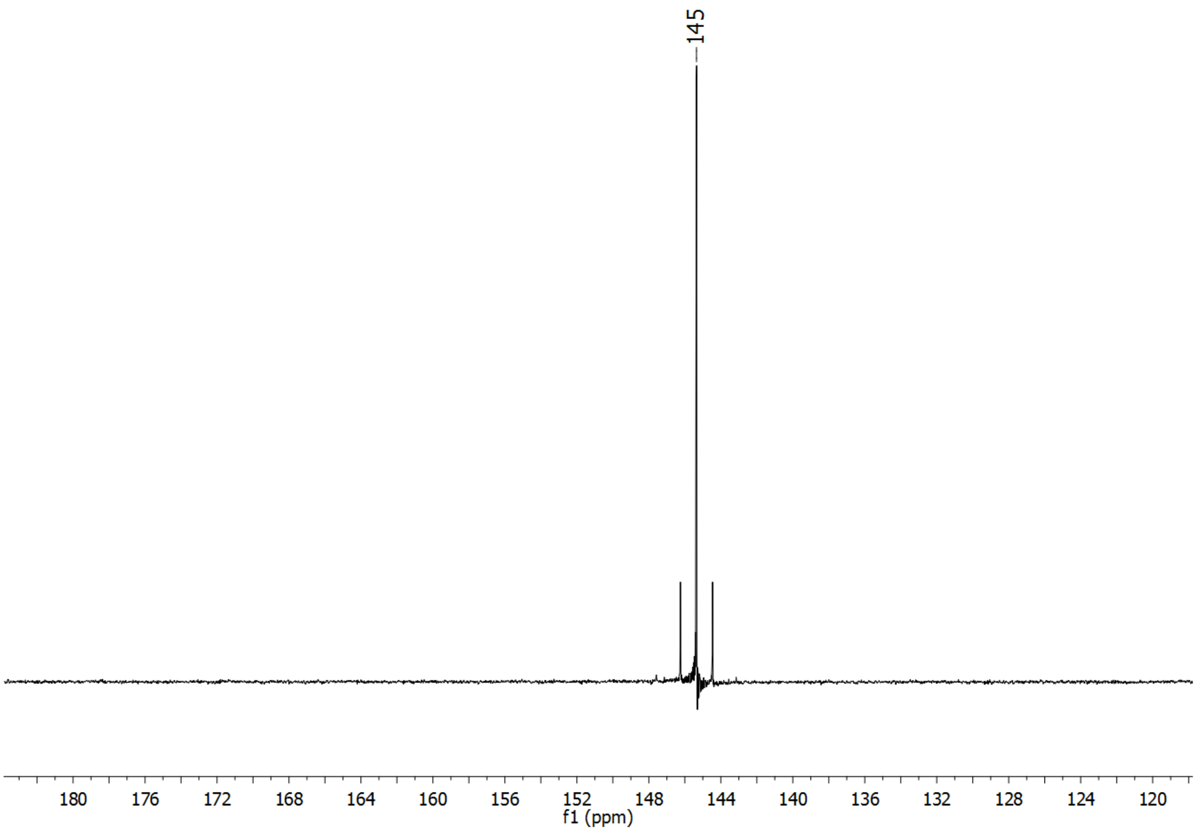

Figure S29: ^{119}Sn NMR spectrum of **8** (112 MHZ, 25°C, CD_2Cl_2).

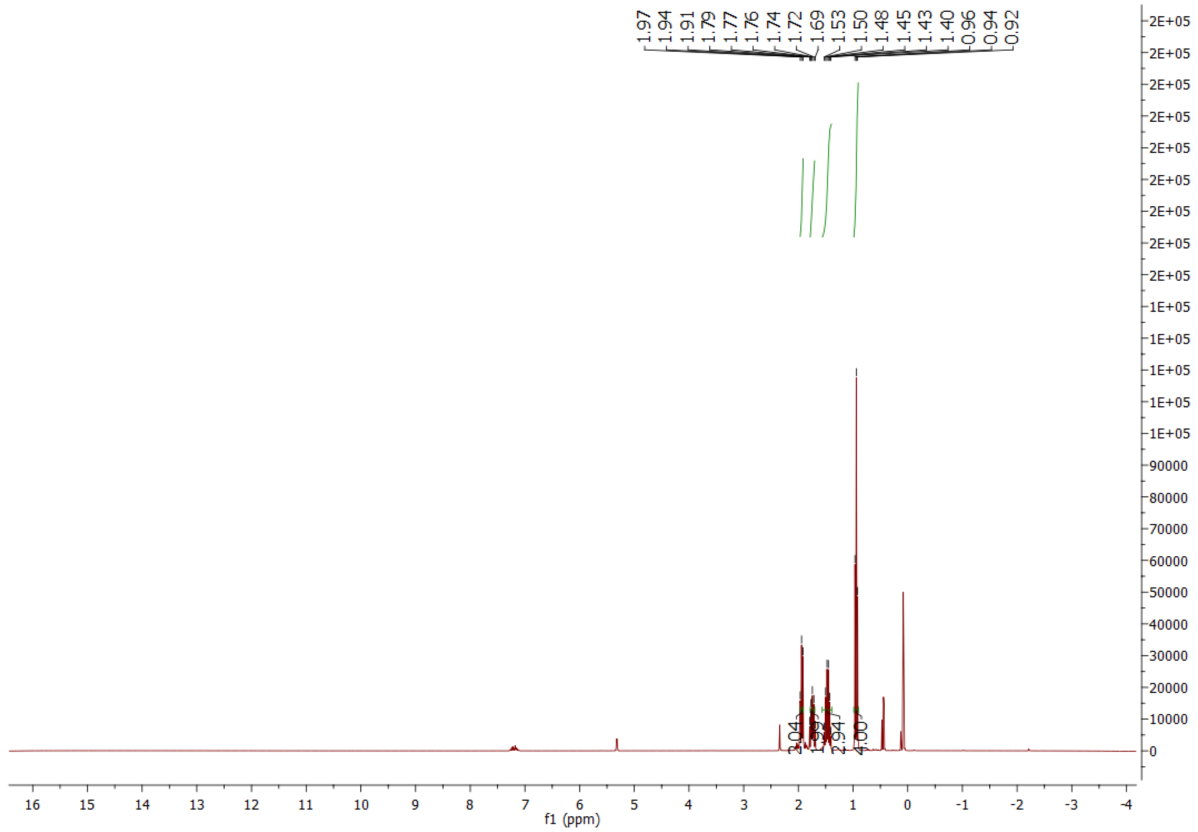

Figure S30: ^{1}H NMR spectrum of **9** (300 MHZ, 25°C, CD_2Cl_2).

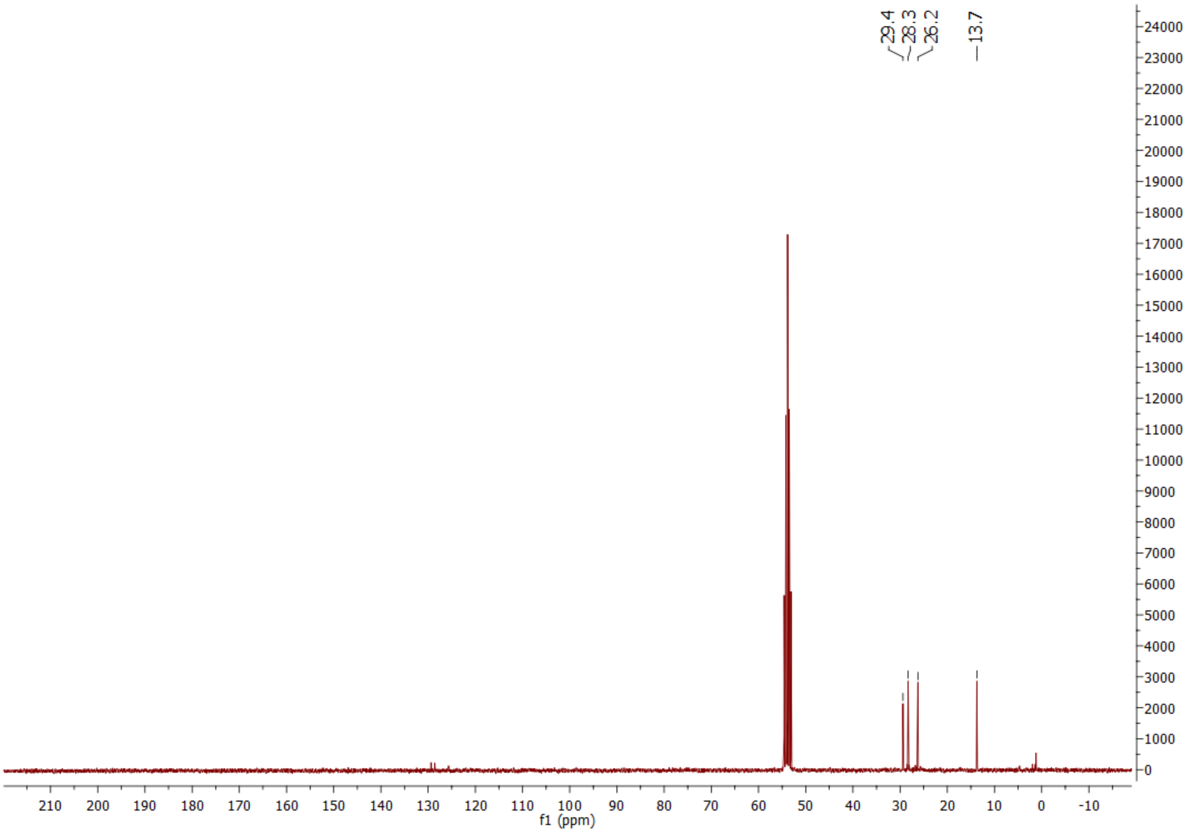

Figure S31: ^{13}C NMR spectrum of **9** (300 MHZ, 25°C, CD_2Cl_2).

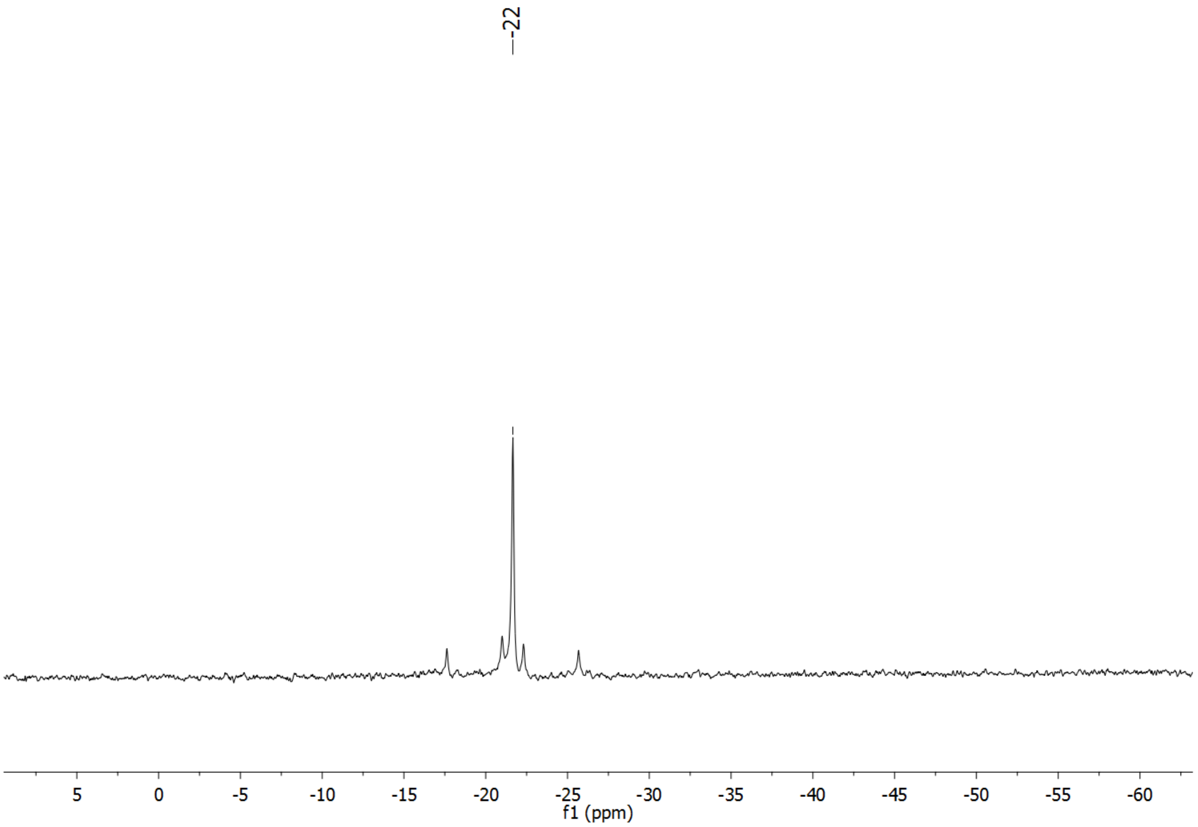

Figure S32: ^{119}Sn NMR spectrum of **9** (187 MHZ, 25°C, CD_2Cl_2).

3. IR Spectra

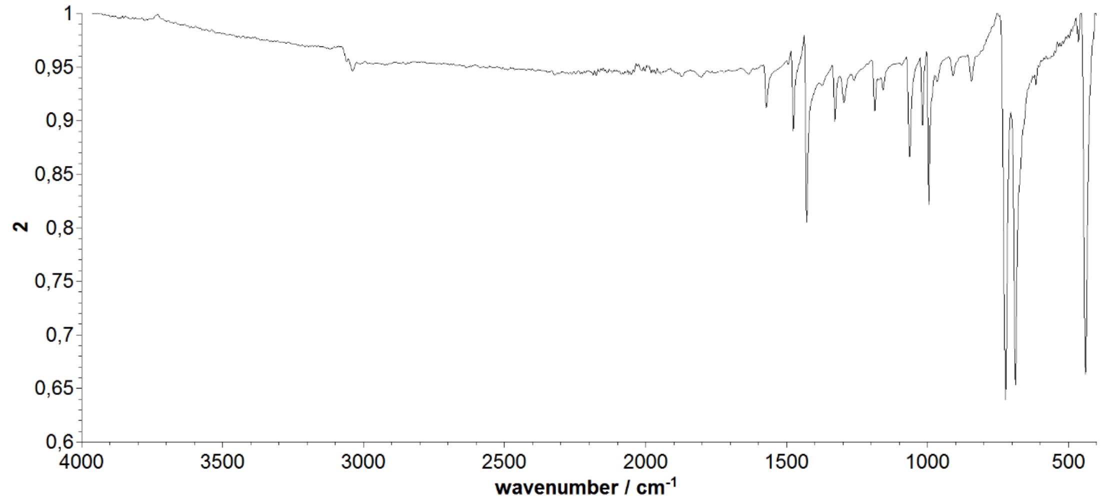

Figure S33: IR spectrum of **1**.

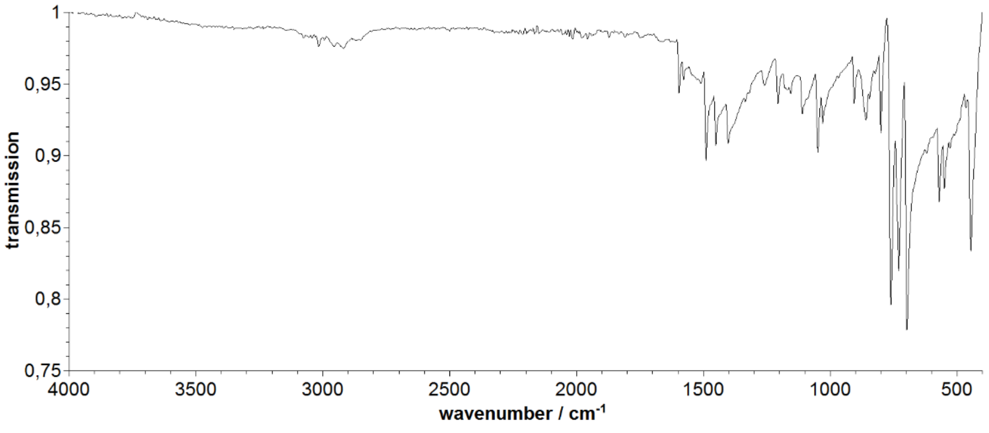

Figure S34: IR spectrum of **2**.

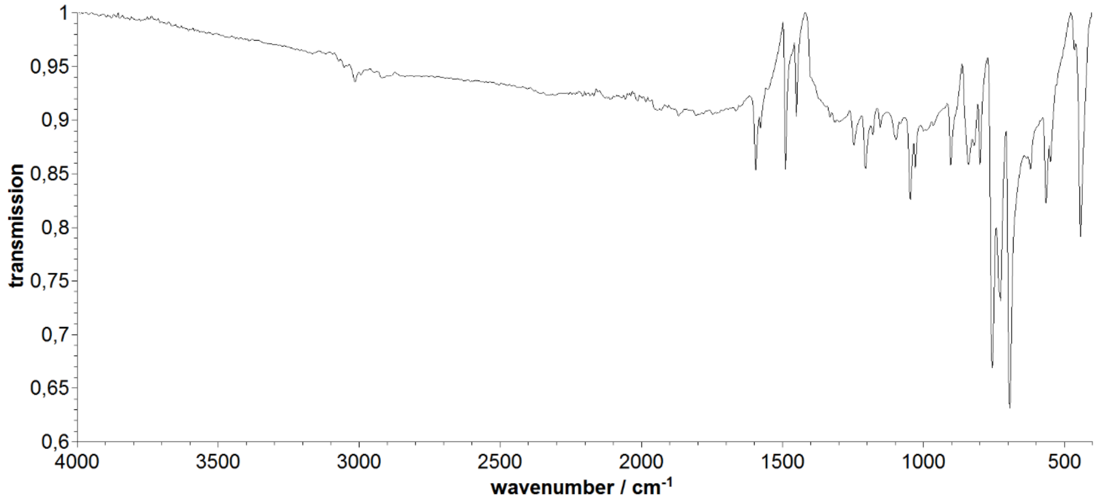

Figure S35: IR spectrum of **3**.

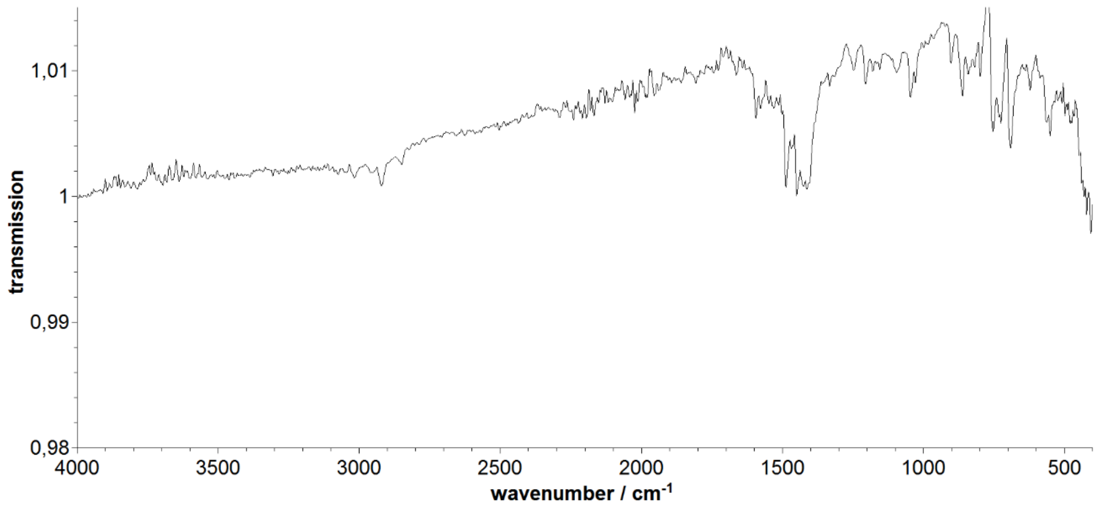

Figure S36: IR spectrum of **4**.

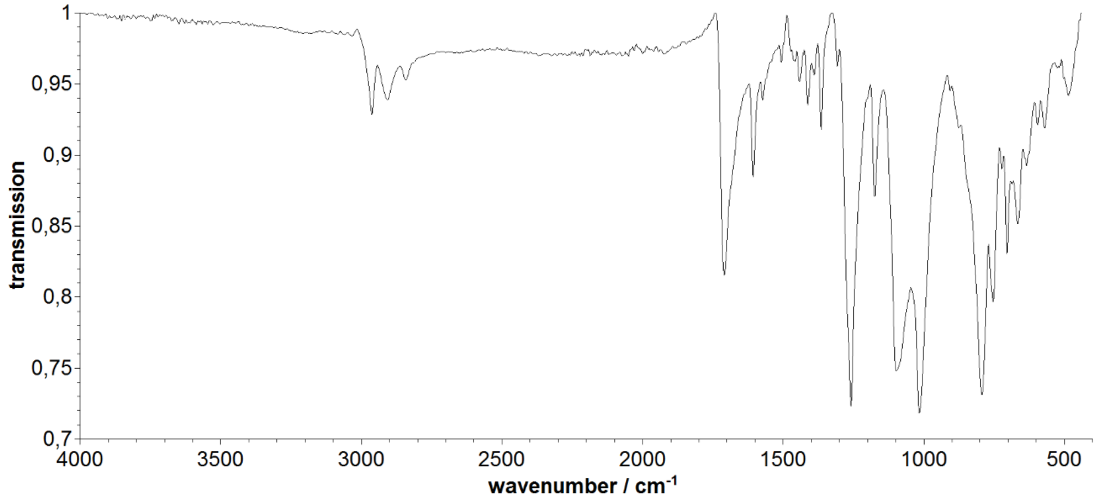

Figure S37: IR spectrum of **5**.

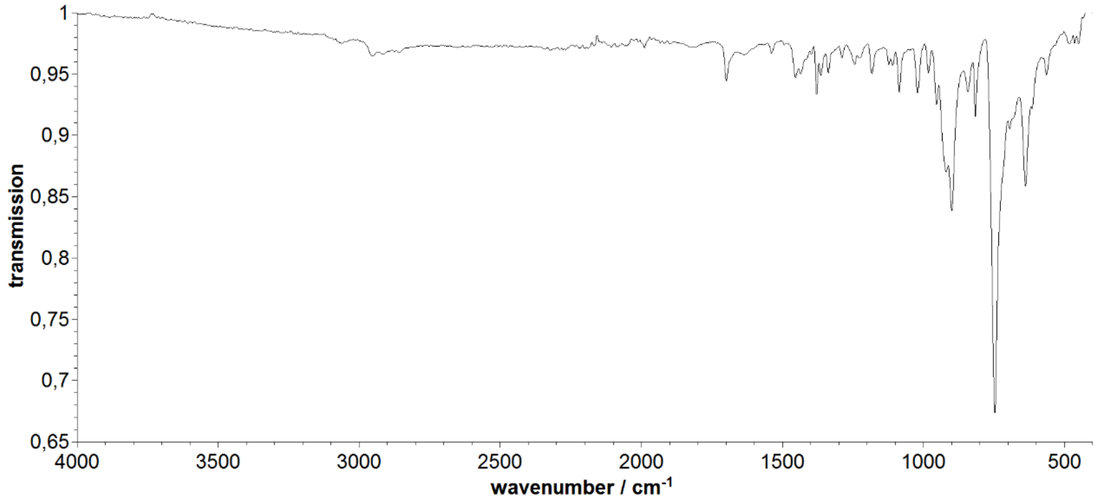

Figure S38: IR spectrum of **6**.

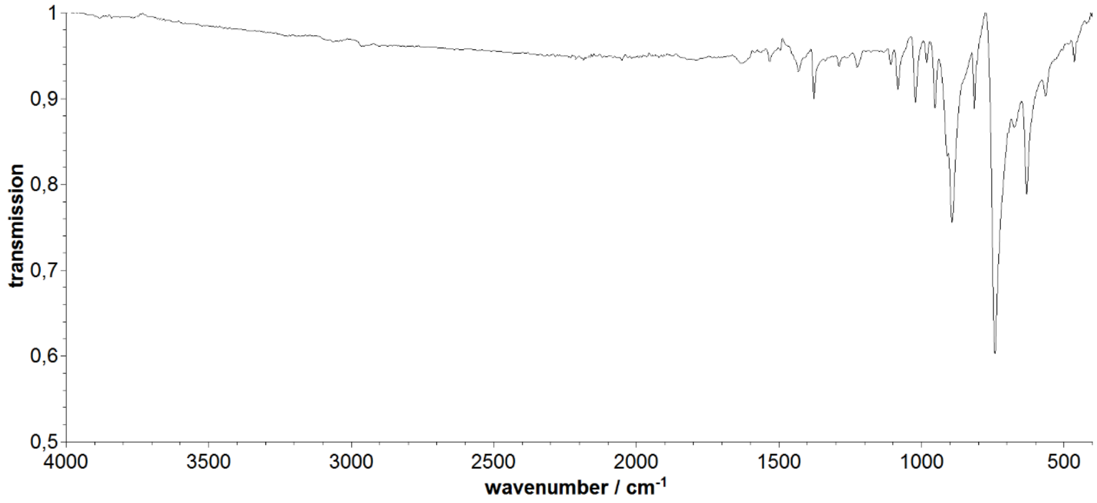

Figure S39: IR spectrum of **7**.

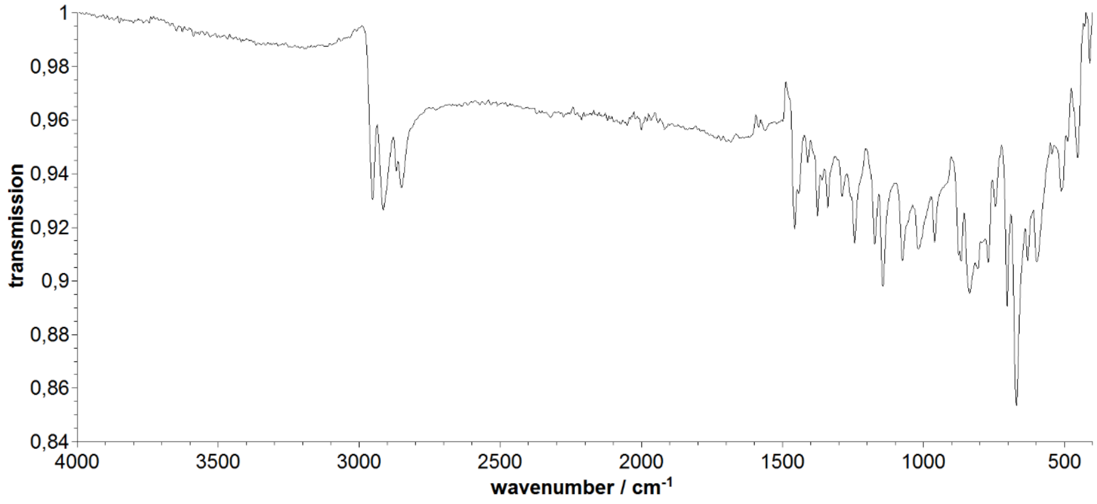

Figure S40: IR spectrum of **8**.

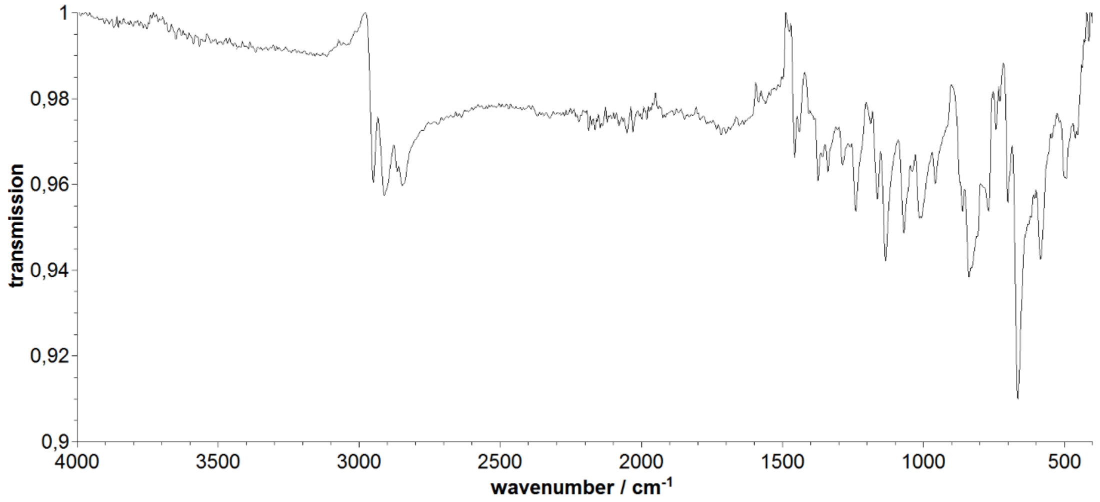

Figure S41: IR spectrum of **9**

4. X-Ray Powder Diffraction

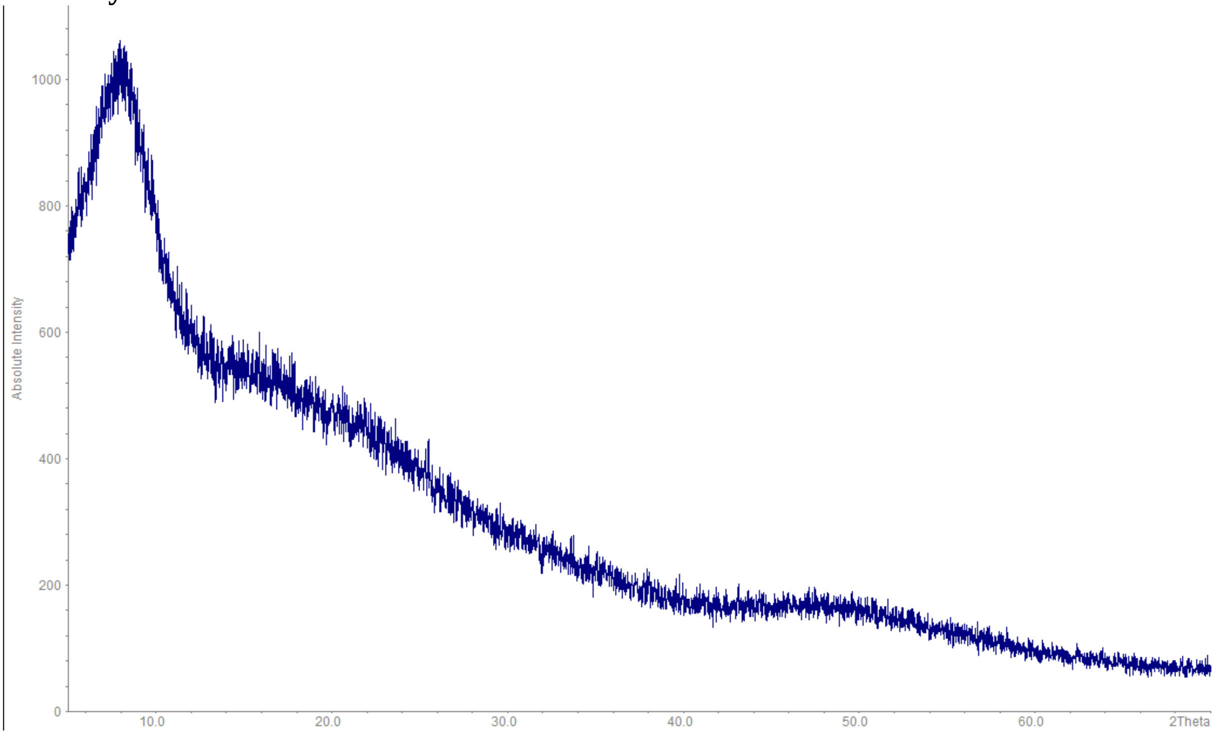

Figure S42: X-Ray powder diffractogram of **1**.

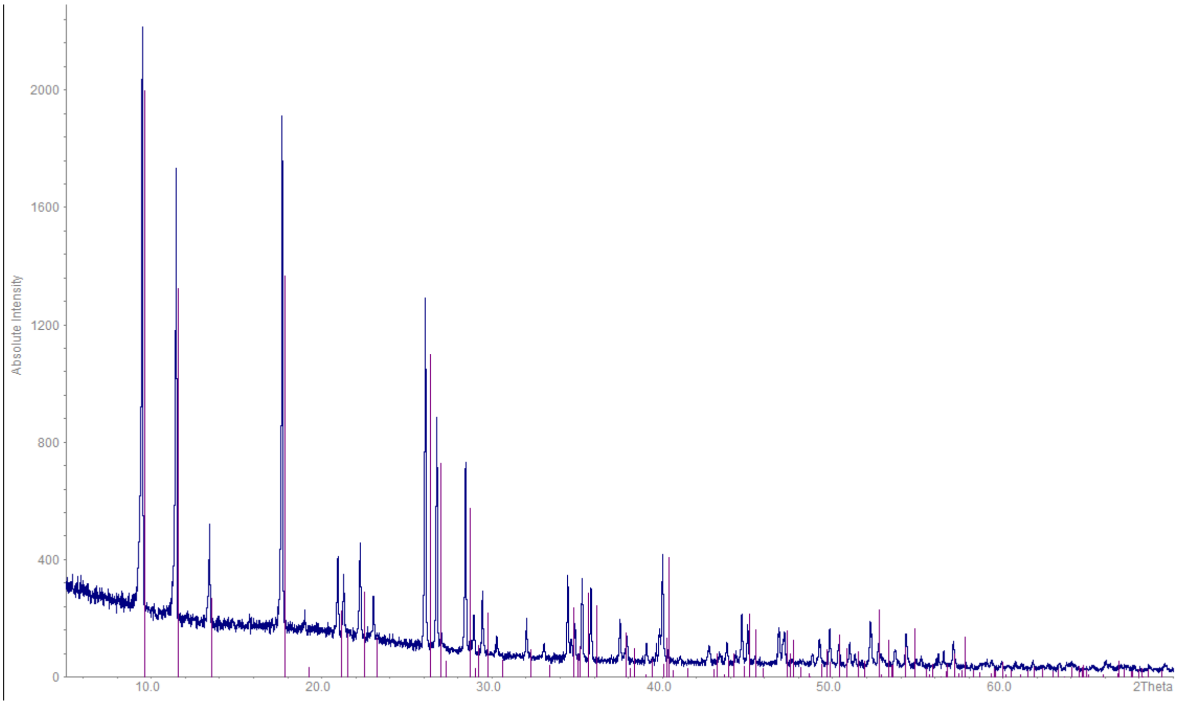

Figure S43: X-Ray powder diffractogram of **2**, compared with a simulated diffractogram obtained from the crystal structure of **2**. The angular shift arises from the temperature difference between powder (room temperature) and single-crystal measurement (100 K).

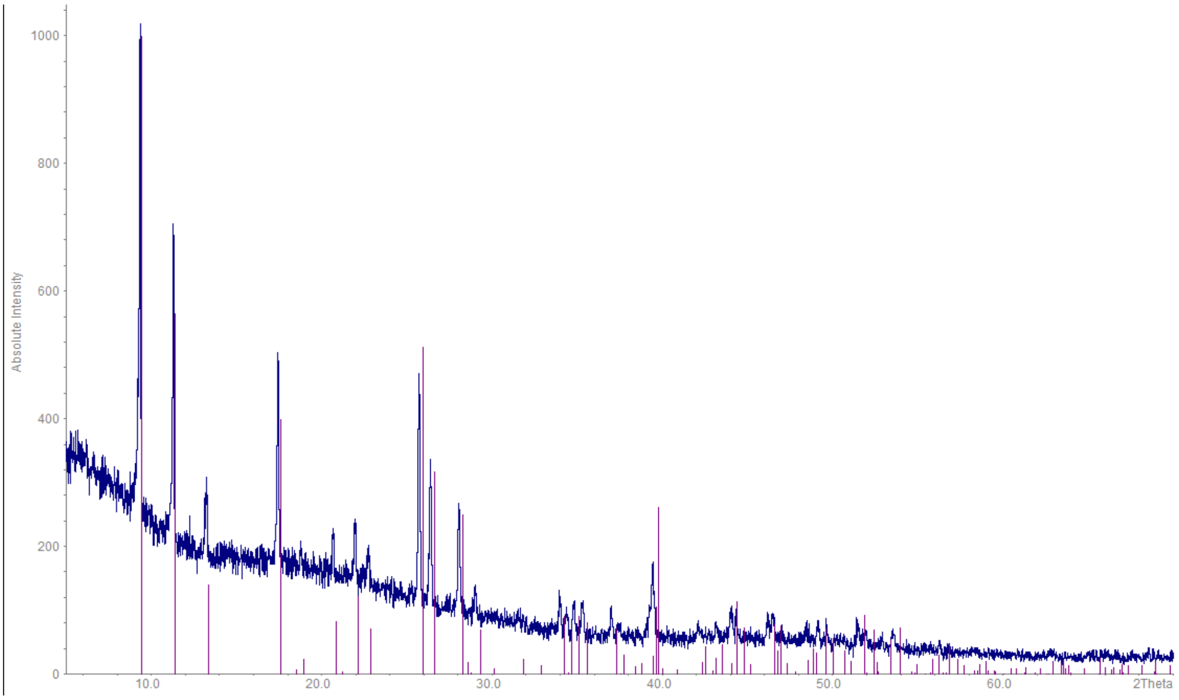

Figure S44: X-Ray powder diffractogram of **3**, compared with a simulated diffractogram obtained from the crystal structure of **3**. The angular shift arises from the temperature difference between powder (room temperature) and single-crystal measurement (100 K).

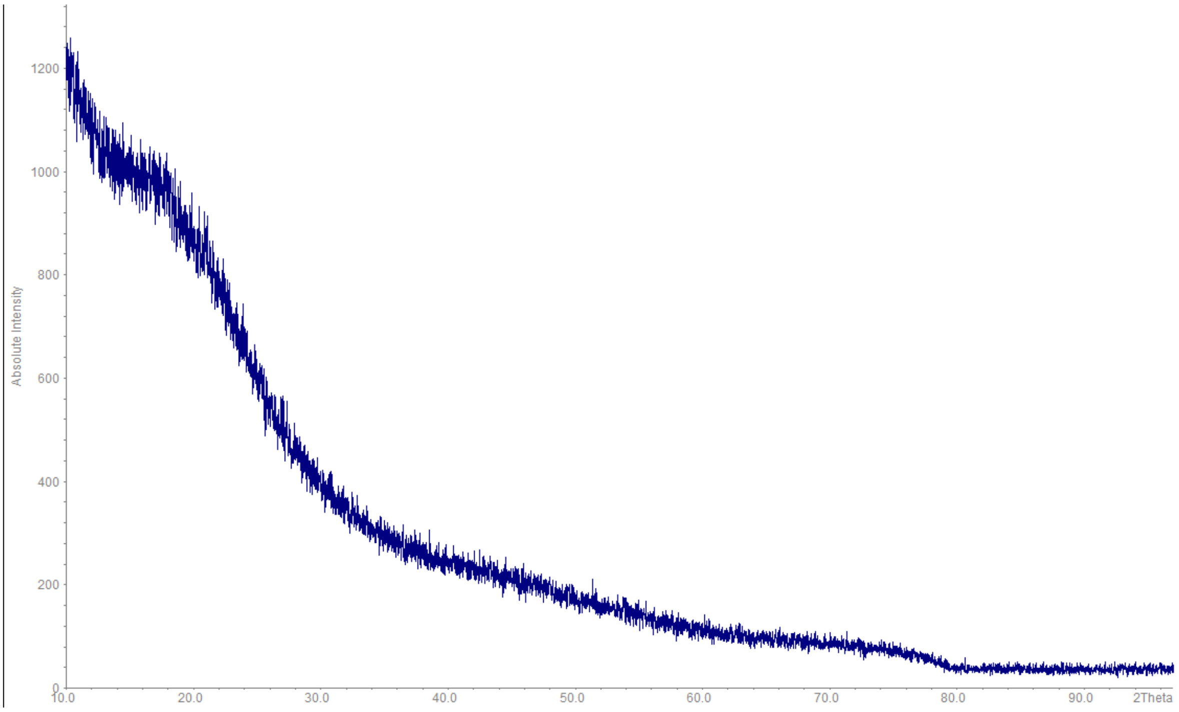

Figure S45: X-Ray powder diffractogram of **4**.

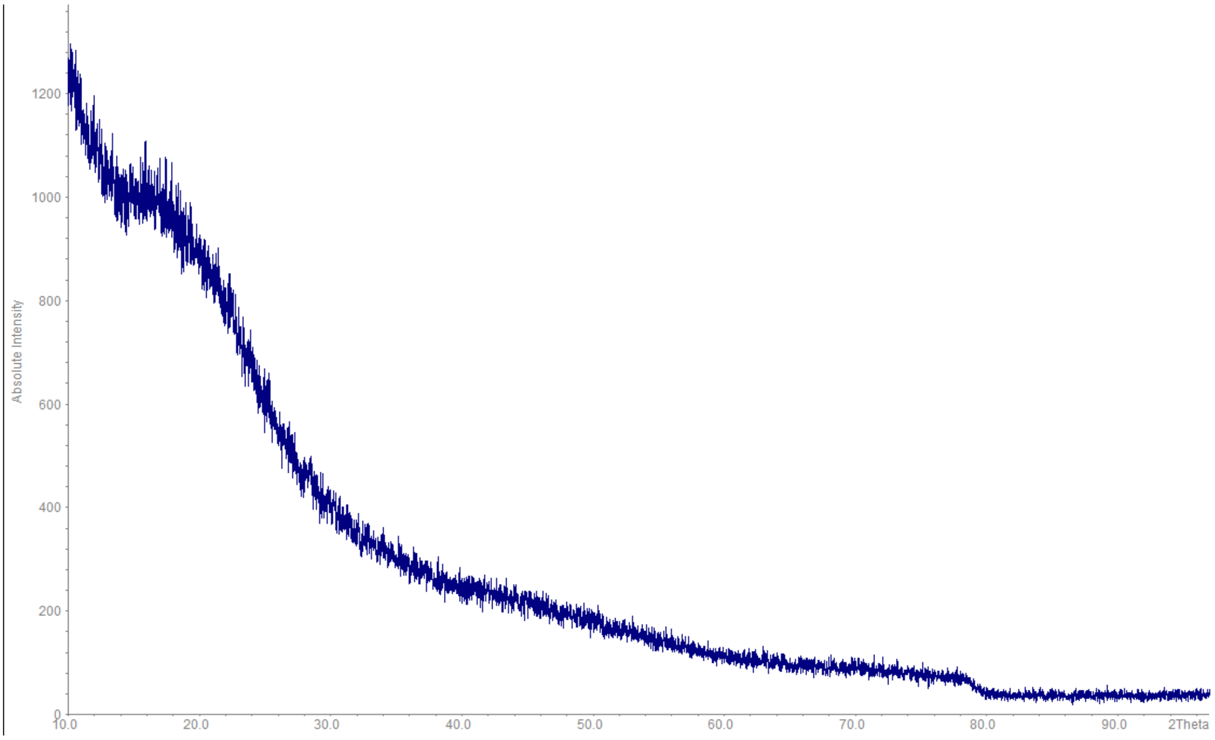

Figure S46: X-Ray powder diffractogram of **5**.

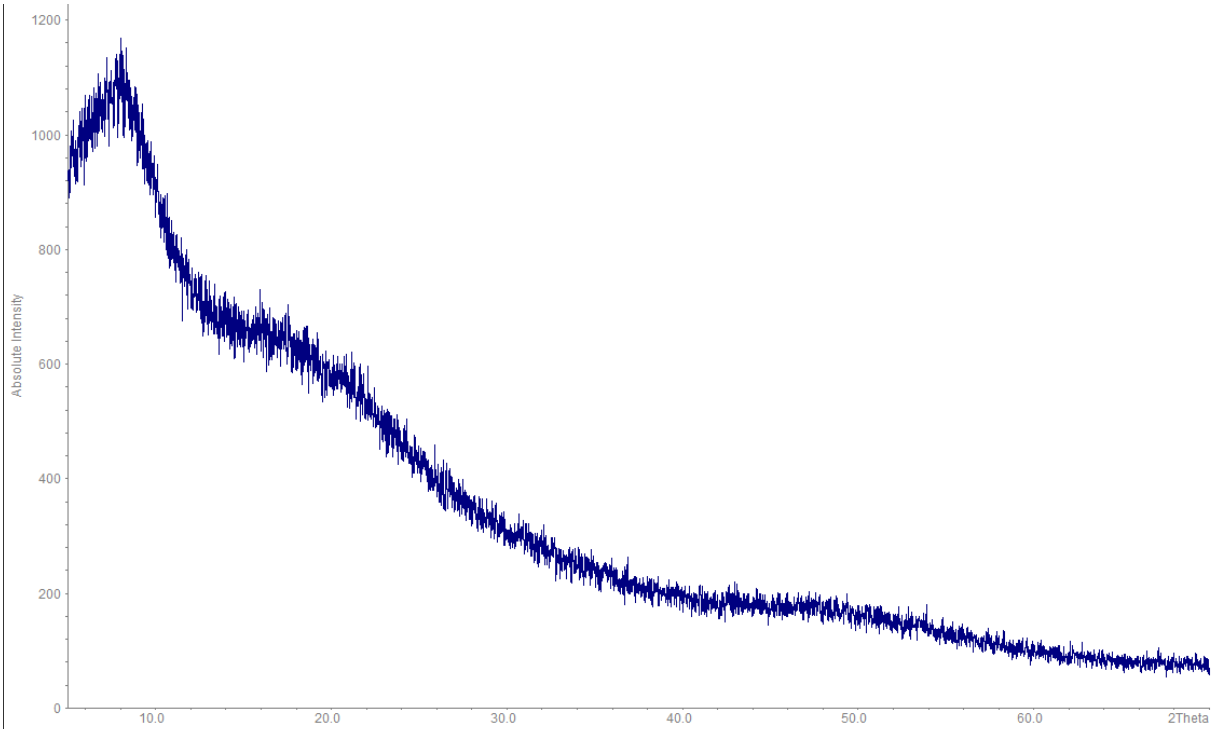

Figure S47: X-Ray powder diffractogram of **6**.

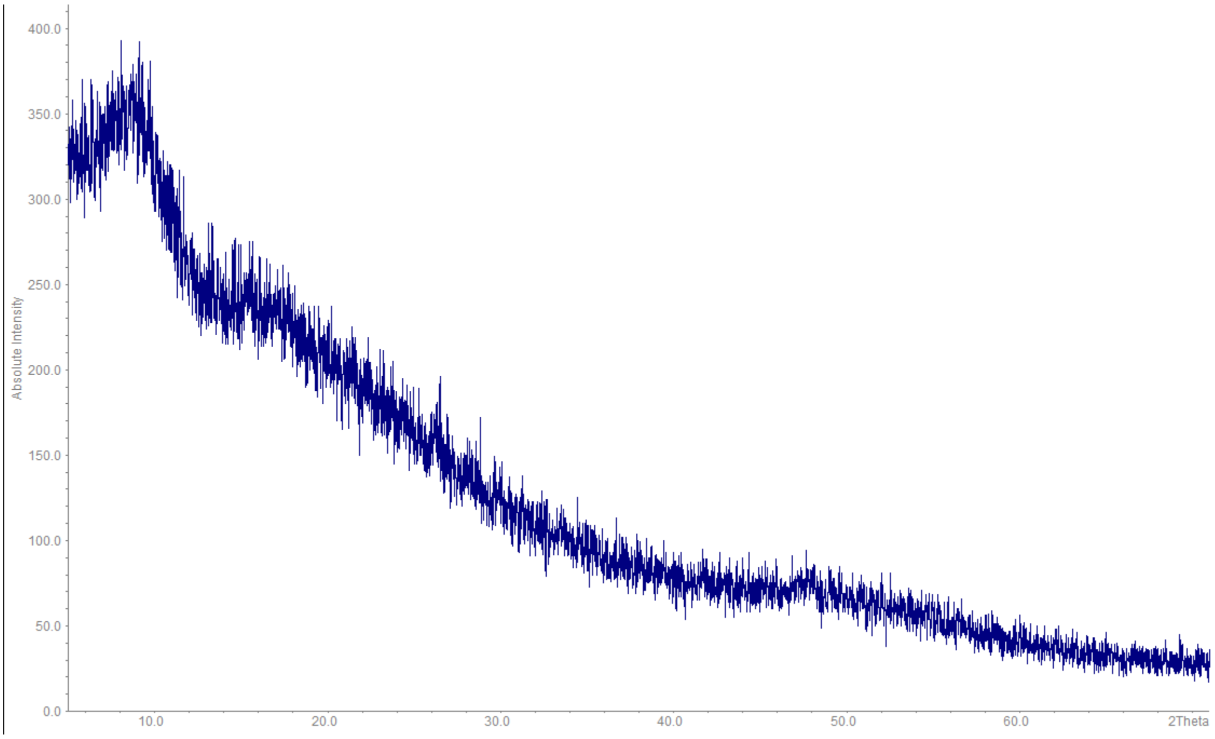

Figure S48: X-Ray powder diffractogram of **7**.

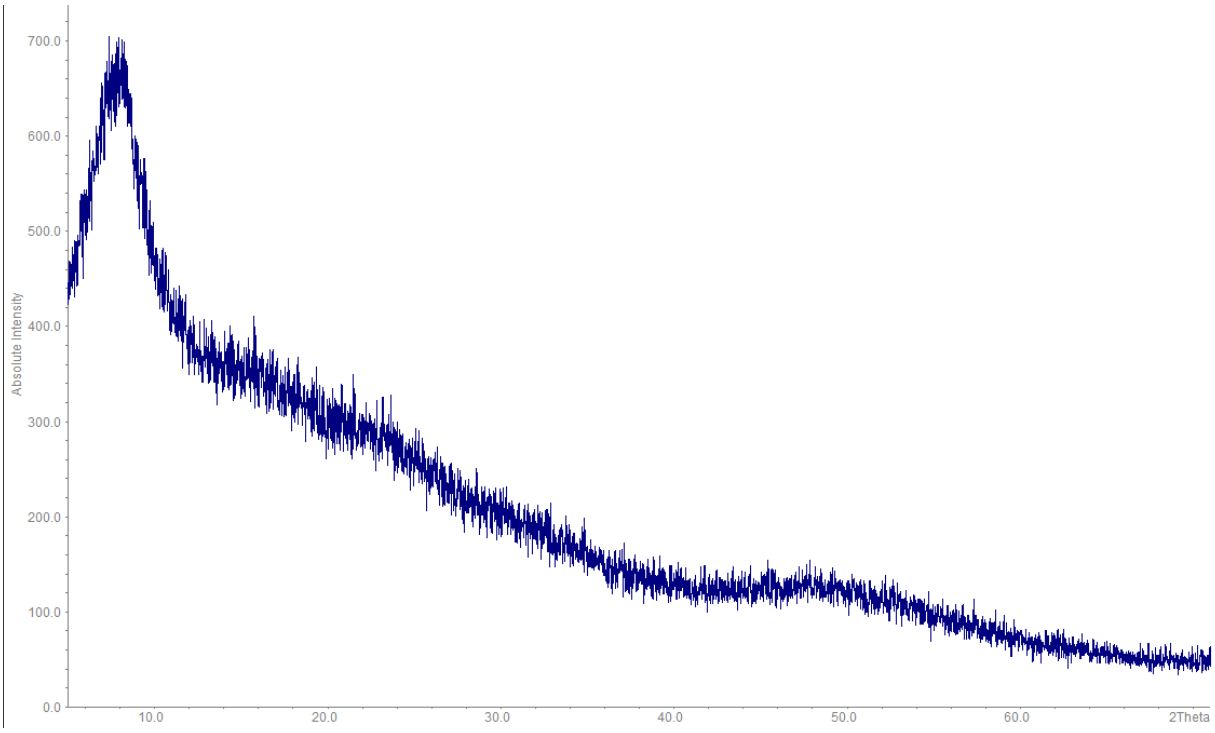

Figure S49: X-Ray powder diffractogram of **8**.

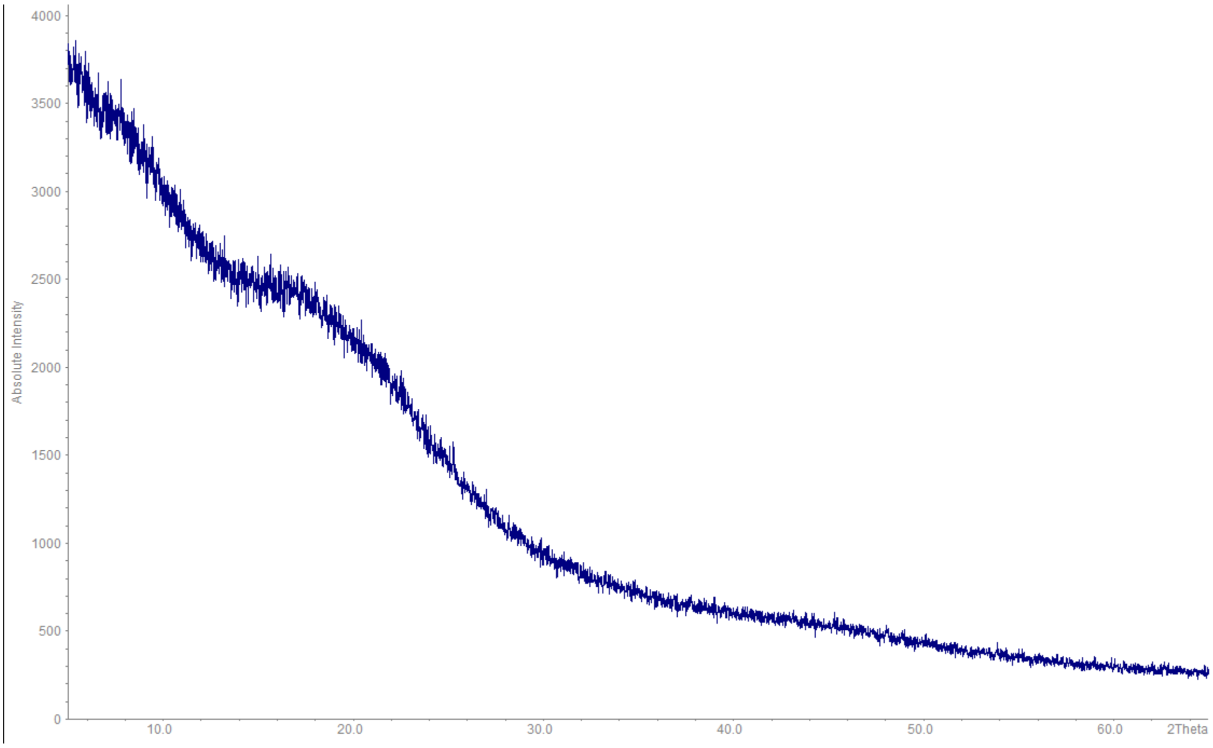

Figure S50: X-Ray powder diffractogram of **9**.

5. Single-Crystal X-Ray Structure of Compound 2

Figure S51 shows a cut-out of the crystal structure of compound **2**, as determined by means of single-crystal X-ray diffraction. Tables S1-S3 provide supplementary X-ray crystallographic data for compound **2.**

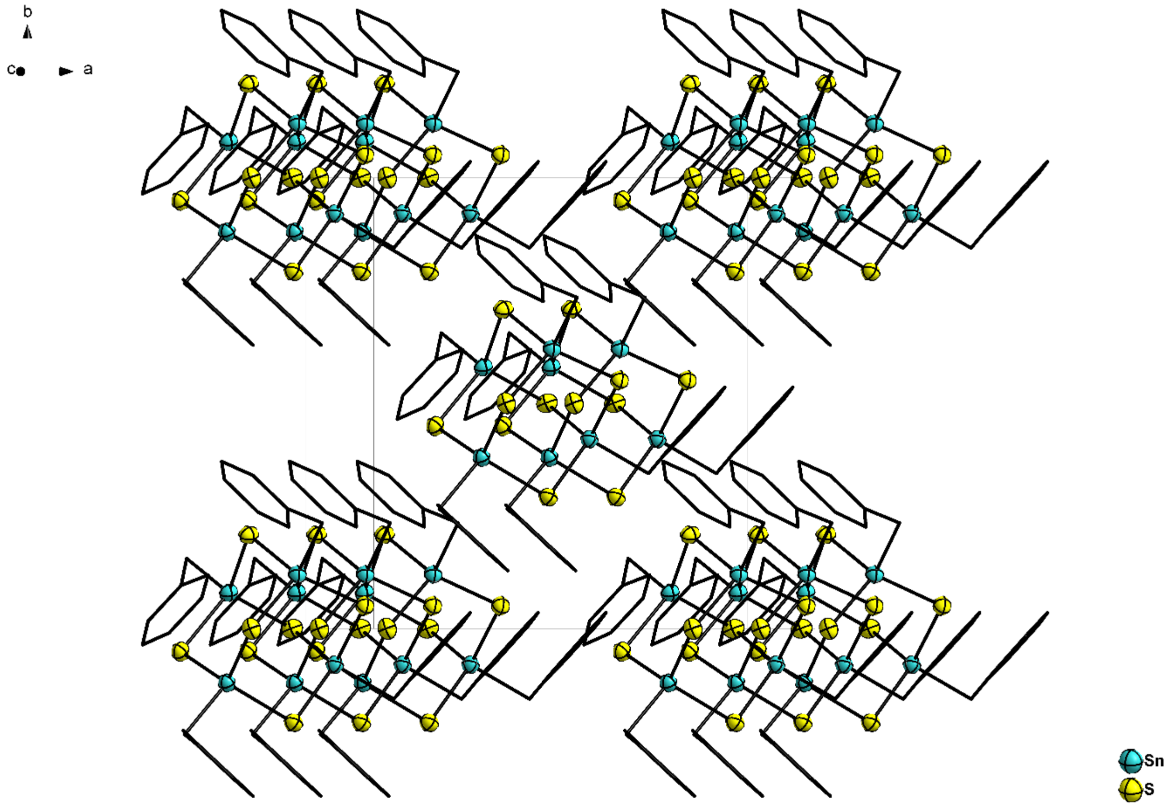

Figure S51: Crystal structure of **2**, showing the ordering of the molecules along the crystallographic *c* axis. Ellipsoids are drawn at 50% probability, hydrogen atoms are omitted for clarity.

Table S1: Crystal data and structure refinement for **2**.

Empirical formula	$C_{28}H_{28}S_6Sn_4$	
Formula weight	1031.62	
Temperature	100(2) K	
Wavelength	1.54178 Å	
Crystal system	Tetragonal	
Space group	$I\bar{4}$	
Unit cell dimensions	*a* = 13.0446(3) Å	α = 90°.
	b = 13.0446(3) Å	β = 90°.
	c = 9.4366(4) Å	γ = 90°.
Volume	1605.75(10) Å^3	
Z	2	
Density (calculated)	2.134 Mg/m^3	
Absorption coefficient	28.243 mm^{-1}	
F(000)	984	
Crystal size	0.15 x 0.13 x 0.10 mm^3	
Theta range for data collection	4.794 to 71.972°.	
Index ranges	-14<=h<=16, -16<=k<=14, -10<=l<=11	
Reflections collected	12918	
Independent reflections	1553 [R(int) = 0.0570]	
Completeness to theta = 67.679°	100.0 %	
Absorption correction	Sphere	
Max. and min. transmission	0.0105 and 0.0014	
Refinement method	Full-matrix least-squares on F2	
Data / restraints / parameters	1553 / 0 / 87	
Goodness-of-fit on F2	0.832	
Final R indices [I>2sigma(I)]	R1 = 0.0292, wR2 = 0.0869	
R indices (all data)	R1 = 0.0296, wR2 = 0.0877	
Absolute structure parameter	-0.025(17)	
Largest diff. peak and hole	0.776 and -0.467 e.Å-3	

Table S2: Atomic coordinates (x 104) and equivalent isotropic displacement parameters (Å2x 103) for **2.** U(eq) is defined as one third of the trace of the orthogonalized Uij tensor.

	x	y	z	U(eq)
Sn(01)	5798(1)	6202(1)	1440(1)	26(1)
S(002)	4492(2)	7086(2)	117(2)	30(1)
S(003)	5000	5000	3009(3)	30(1)
C(004)	6191(7)	7258(6)	5295(10)	34(2)
C(005)	4644(6)	8711(6)	5087(11)	37(2)
C(006)	6559(6)	7346(6)	2707(9)	28(2)
C(007)	5960(6)	7663(5)	3964(9)	32(2)
C(008)	5681(6)	7587(6)	6485(11)	37(2)
C(009)	4909(7)	8309(7)	6395(11)	42(2)
C(00A)	5169(6)	8398(6)	3871(9)	33(2)

Table S3: Bond lengths [Å] and angles [°] for **2**.

Sn(01)-C(006)	2.155(8)	Sn(01)-S(003)-Sn(01)#3	103.60(11)
Sn(01)-S(003)	2.3948(18)	C(008)-C(004)-C(007)	120.8(8)
Sn(01)-S(002)	2.405(2)	C(008)-C(004)-H(004)	119.6
Sn(01)-S(002)#1	2.4086(19)	C(007)-C(004)-H(004)	119.6
C(004)-C(008)	1.375(13)	C(009)-C(005)-C(00A)	120.0(7)
C(004)-C(007)	1.395(13)	C(009)-C(005)-H(005)	120.0
C(004)-H(004)	0.9300	C(00A)-C(005)-H(005)	120.0
C(005)-C(009)	1.385(15)	C(007)-C(006)-Sn(01)	113.3(5)
C(005)-C(00A)	1.396(13)	C(007)-C(006)-H(00A)	108.9
C(005)-H(005)	0.9300	Sn(01)-C(006)-H(00A)	108.9
C(006)-C(007)	1.479(12)	C(007)-C(006)-H(00B)	108.9
C(006)-H(00A)	0.9700	Sn(01)-C(006)-H(00B)	108.9
C(006)-H(00B)	0.9700	H(00A)-C(006)-H(00B)	107.7
C(007)-C(00A)	1.411(11)	C(004)-C(007)-C(00A)	118.1(8)
C(008)-C(009)	1.381(13)	C(004)-C(007)-C(006)	120.1(7)
C(008)-H(008)	0.9300	C(00A)-C(007)-C(006)	121.8(7)
C(009)-H(009)	0.9300	C(004)-C(008)-C(009)	121.1(10)
C(00A)-H(00C)	0.9300	C(004)-C(008)-H(008)	119.5
C(006)-Sn(01)-S(003)	108.1(2)	C(009)-C(008)-H(008)	119.5
C(006)-Sn(01)-S(002)	106.4(2)	C(008)-C(009)-C(005)	119.6(9)
S(003)-Sn(01)-S(002)	109.10(5)	C(008)-C(009)-H(009)	120.2
C(006)-Sn(01)-S(002)#1	106.1(2)	C(005)-C(009)-H(009)	120.2
S(003)-Sn(01)-S(002)#1	115.71(6)	C(005)-C(00A)-C(007)	120.4(8)
S(002)-Sn(01)-S(002)#1	110.96(4)	C(005)-C(00A)-H(00C)	119.8
Sn(01)-S(002)-Sn(01)#2	104.38(8)	C(007)-C(00A)-H(00C)	119.8

Symmetry transformations used to generate equivalent atoms:

#1 y,-x+1,-z   #2 -y+1,x,-z   #3 -x+1,-y+1,z

6. Single-Crystal X-Ray Crystallography of Compound 3

Figure S52 shows a cut-out of the crystal structure of compound **3**, as determined by means of single-crystal X-ray diffraction. Tables S4-S6 provide supplementary X-ray crystallographic data for compound **3.**

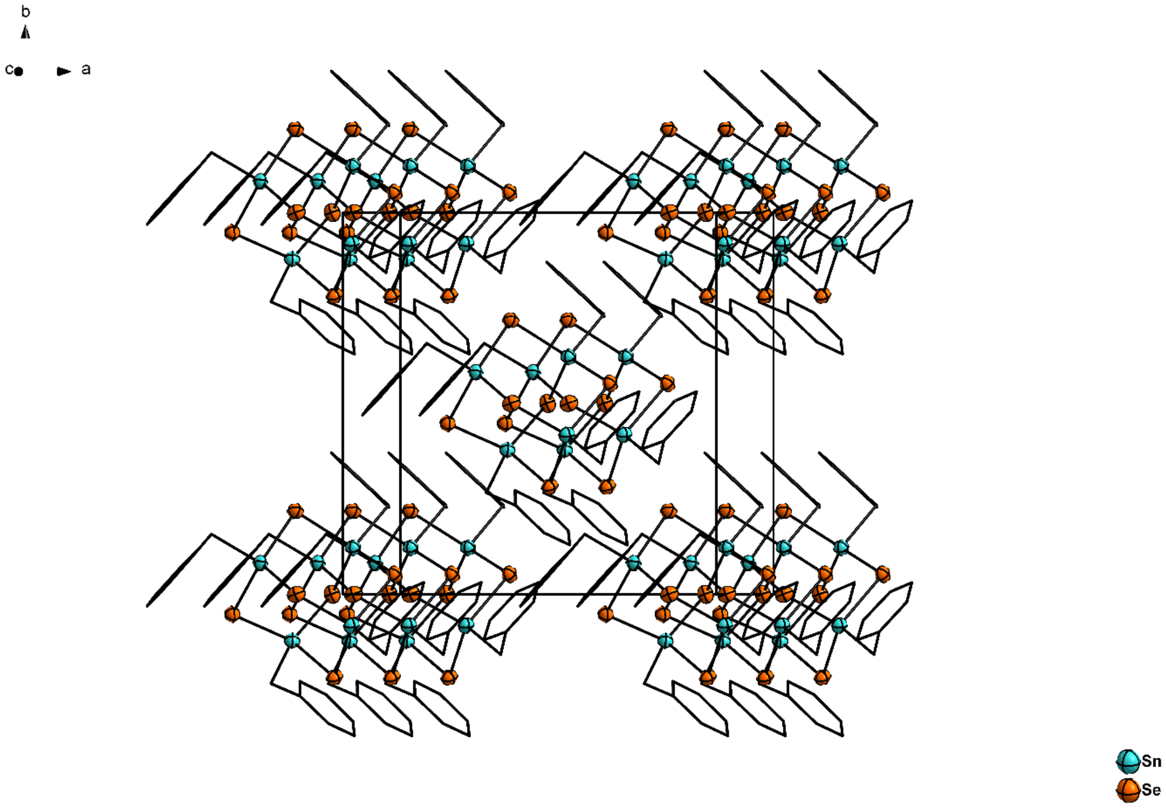

Figure S52: Crystal structure of **3**, showing the ordering of the molecules along the crystallographic *c* axis. Ellipsoids are drawn at 50% probability, hydrogen atoms are omitted for clarity.

Table S4: Crystal data and structure refinement for **3**.

Empirical formula	$C_{28}H_{28}Se_6Sn_4$
Formula weight	1313.02
Temperature	100(2) K
Wavelength	1.54178 Å
Crystal system	Tetragonal
Space group	$I\bar{4}$
Unit cell dimensions	a = 13.24260(10) Å α = 90°.
	b = 13.24260(10) Å β = 90°.
	c = 9.5754(2) Å γ = 90°.
Volume	1679.20(4) Å^3
Z	2
Density (calculated)	2.597 Mg/m^3
Absorption coefficient	30.959 mm^{-1}
F(000)	1200
Crystal size	0.25 x 0.20 x 0.15 mm^3
Theta range for data collection	4.722 to 71.461°.
Index ranges	-16<=h<=10, -16<=k<=15, -11<=l<=11
Reflections collected	14136
Independent reflections	1625 [R(int) = 0.0365]
Completeness to theta = 67.679°	100.0 %
Absorption correction	Sphere
Max. and min. transmission	0.0005 and 0.0000
Refinement method	Full-matrix least-squares on F2
Data / restraints / parameters	1625 / 0 / 87
Goodness-of-fit on F2	0.879
Final R indices [I>2sigma(I)]	R1 = 0.0318, wR2 = 0.0910
R indices (all data)	R1 = 0.0319, wR2 = 0.0912
Absolute structure parameter	-0.015(7)
Largest diff. peak and hole	1.581 and -1.310 e.Å-3

Table S5: Atomic coordinates (x 104) and equivalent isotropic displacement parameters (Å2x 103) for **3**. U(eq) is defined as one third of the trace of the orthogonalized Uij tensor.

	x	y	z	U(eq)
Sn(01)	5818(1)	6219(1)	8541(1)	28(1)
Se(02)	5000	5000	6862(1)	32(1)
Se(03)	4476(1)	7177(1)	9872(1)	31(1)
C(004)	4690(7)	8706(7)	4994(12)	41(2)
C(005)	4934(8)	8326(8)	3696(13)	45(2)
C(006)	6195(7)	7278(7)	4748(11)	39(2)
C(007)	5202(7)	8386(6)	6184(11)	37(2)
C(008)	6576(6)	7338(7)	7291(9)	31(2)
C(009)	5983(6)	7668(6)	6059(10)	33(2)
C(00A)	5682(8)	7620(8)	3580(11)	42(2)

Table S6: Bond lengths [Å] and angles [°] for **3**.

Sn(01)-C(008)	2.153(9)
Sn(01)-Se(02)	2.5227(9)
Sn(01)-Se(03)#1	2.5283(9)
Sn(01)-Se(03)	2.5292(10)
C(004)-C(005)	1.379(16)
C(004)-C(007)	1.392(14)
C(004)-H(004)	0.9300
C(005)-C(00A)	1.366(16)
C(005)-H(005)	0.9300
C(006)-C(00A)	1.384(16)
C(006)-C(009)	1.386(14)
C(006)-H(006)	0.9300
C(007)-C(009)	1.410(12)
C(007)-H(007)	0.9300
C(008)-C(009)	1.483(12)
C(008)-H(00A)	0.9700
C(008)-H(00B)	0.9700
C(00A)-H(00C)	0.9300
C(008)-Sn(01)-Se(02)	106.7(2)
C(008)-Sn(01)-Se(03)#1	104.6(2)
Se(02)-Sn(01)-Se(03)#1	117.13(3)
C(008)-Sn(01)-Se(03)	105.2(2)
Se(02)-Sn(01)-Se(03)	109.89(3)
Se(03)#1-Sn(01)-Se(03)	112.32(2)
Sn(01)-Se(02)-Sn(01)#2	100.82(5)
Sn(01)#3-Se(03)-Sn(01)	101.64(4)
C(005)-C(004)-C(007)	120.8(9)
C(005)-C(004)-H(004)	119.6
C(007)-C(004)-H(004)	119.6
C(00A)-C(005)-C(004)	119.5(10)
C(00A)-C(005)-H(005)	120.2
C(004)-C(005)-H(005)	120.2
C(00A)-C(006)-C(009)	120.7(9)
C(00A)-C(006)-H(006)	119.6
C(009)-C(006)-H(006)	119.6
C(004)-C(007)-C(009)	119.6(9)
C(004)-C(007)-H(007)	120.2
C(009)-C(007)-H(007)	120.2
C(009)-C(008)-Sn(01)	113.4(6)
C(009)-C(008)-H(00A)	108.9
Sn(01)-C(008)-H(00A)	108.9
C(009)-C(008)-H(00B)	108.9
Sn(01)-C(008)-H(00B)	108.9
H(00A)-C(008)-H(00B)	107.7
C(006)-C(009)-C(007)	118.4(9)
C(006)-C(009)-C(008)	120.3(8)
C(007)-C(009)-C(008)	121.3(9)
C(005)-C(00A)-C(006)	120.9(11)
C(005)-C(00A)-H(00C)	119.5
C(006)-C(00A)-H(00C)	119.5

Symmetry transformations used to generate equivalent atoms:

#1 y,-x+1,-z+2 #2 -x+1,-y+1,z #3 -y+1,x,-z+2

7. Lowest Singlet Excitation Energies

Table S7: Lowest singlet excitation energies of compounds **A** and **1 – 9**, as calculated by means of time-dependent density functional theory (TD-DFT) calculations.

Compound	Energy / eV
**A**	3.81
**1**	3.24
**2**	3.66
**3**	3.17
**4**	3.85
**5**	3.29
**6**	3.00
**7**	2.77
**8**	3.81
**9**	3.26

8. UV-Vis-Absorption Spectra

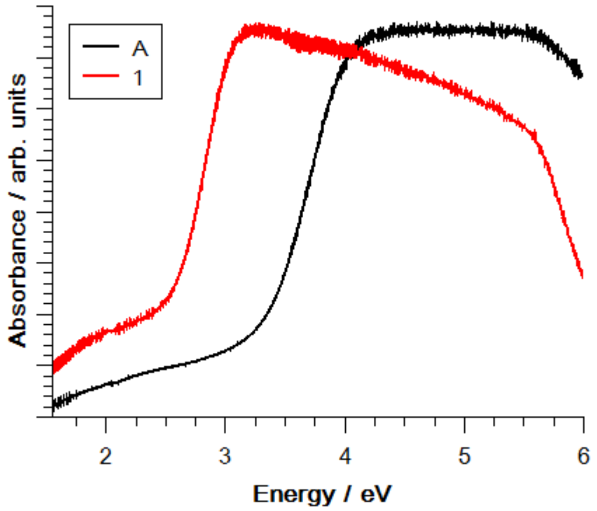

Figure S53: UV-Vis absorption spectra of **A** and **1**, indicating an onset of absorption between approx. 3.2 and 3.9 eV (**A**), and between 2.4 and 3.0 eV (**1**), respectively.

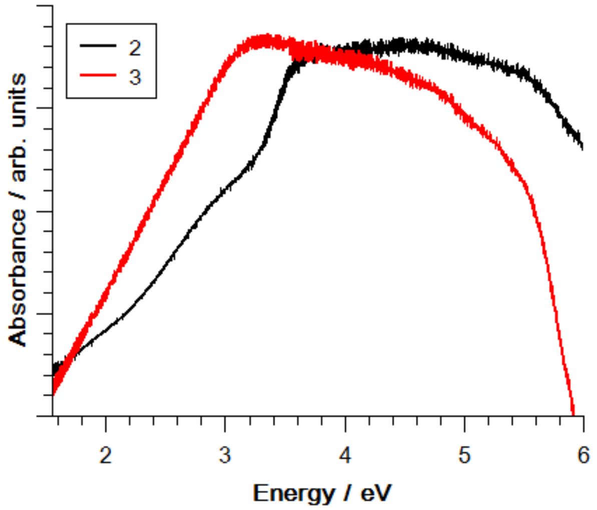

Figure S54: UV-Vis absorption spectra of **2** and **3**, indicating a (broad) onset of absorption between approx. 2.0 and 3.5 eV (**A**), and between 1.6 and 3.0 eV (**1**), respectively.

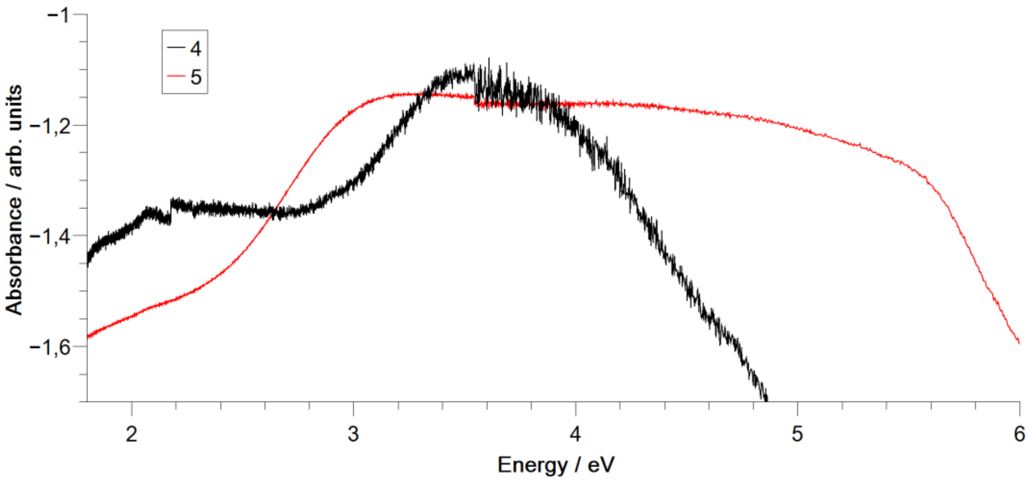

Figure S55: UV-Vis absorption spectra of **4** and **5**, indicating an onset of absorption between approx. 2.9 and 3.4 eV (**4**), and between 2.4 and 2.9 eV (**5**), respectively. The onset in the UV region is an artefact of the spectrometer.

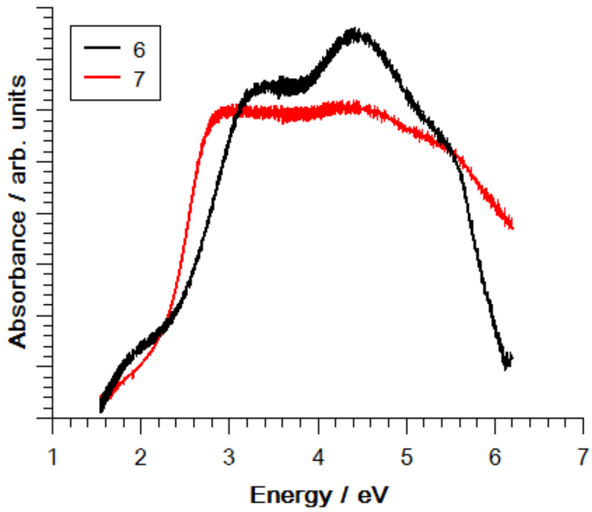

Figure S56: UV-Vis absorption spectra of **6** and **7**, indicating an onset of absorption between approx. 2.4 and 3.0 eV (**6**), and between 2.2 and 2.7 eV (**7**), respectively.

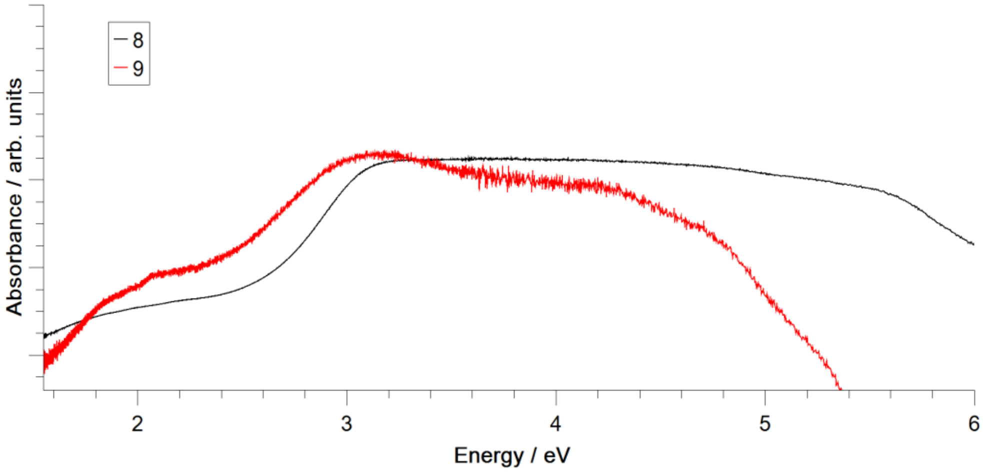

Figure S57: UV-Vis absorption spectra of **8** and **9**, indicating an onset of absorption between approx. 2.6 and 3.0 eV (**8**), and between 2.4 and 2.9 eV (**9**), respectively.
